# Systematic phenotype and genotype characterization of Moebius syndrome

**DOI:** 10.1016/j.gimo.2025.103437

**Published:** 2025-05-19

**Authors:** Bryn D. Webb, Julie A. Jurgens, Narisu Narisu, Zhongyang Zhang, Brenda J. Barry, Carol Van Ryzin, Lori L. Bonnycastle, Wai-Man Chan, Tingfen Yan, Silvio Alessandro Di Gioia, Amy J. Swift, Sarah E. MacKinnon, Darren T. Oystreck, Janet C. Rucker, Tamiesha Frempong, Mary C. Whitman, Edmond J. FitzGibbon, Janice S. Lee, Ke Hao, Caroline Andrews, Monica Erazo, Flavia M. Facio, Sherin Shaaban, Thomas P. Naidich, Peter S. Chines, Tanya J. Lehky, Camilo Toro, Andrea L. Gropman, John A. Butman, Christopher K. Zalewski, Carmen C. Brewer, Audrey Thurm, Joseph Snow, Scott M. Paul, Brian P. Brooks, Carlo Pierpaoli, Caroline D. Robson, David G. Hunter, Francis S. Collins, Ethylin Wang Jabs, Elizabeth C. Engle, Irini Manoli

**Affiliations:** 1 Department of Genetics and Genomic Sciences, Icahn School of Medicine at Mount Sinai, New York, NY; 2 Department of Pediatrics, University of Wisconsin School of Medicine and Public Health, Madison, WI; 3 F.M. Kirby Neurobiology Center, Boston Children’s Hospital, Boston, MA; 4 Department of Neurology, Boston Children’s Hospital, Boston, MA; 5 Department of Neurology, Harvard Medical School, Boston, MA; 6 Broad Institute of MIT and Harvard, Cambridge, MA; 7 Center for Precision Health Research, National Human Genome Research Institute, National Institutes of Health, Bethesda, MD; 8 Howard Hughes Medical Institute, Chevy Chase, MD; 9 Metabolic Medicine Branch, National Human Genome Research Institute, National Institutes of Health, Bethesda, MD; 10 Regeneron Pharmaceuticals, Tarrytown, NY; 11 Department of Ophthalmology, Boston Children’s Hospital, Boston, MA; 12 Department of Ophthalmology, Harvard Medical School, Boston, MA; 13 Eye Clinic, IWK Health Centre, Halifax, NS, Canada; 14 Clinical Vision Science, Faculty of Health, Dalhousie University, Halifax, NS, Canada; 15 Department of Neurology, Icahn School of Medicine at Mount Sinai, New York, NY; 16 Department of Ophthalmology, Icahn School of Medicine at Mount Sinai, New York, NY; 17 Deparment of Neurology, New York University Grossman School of Medicine, New York, NY; 18 Department of Ophthalmology, New York University Grossman School of Medicine, New York, NY; 19 Laboratory of Sensorimotor Research, National Eye Institute, National Institutes of Health, Bethesda, MD; 20 Craniofacial Anomalies and Regeneration Section, National Institute of Dental and Craniofacial Research, National Institutes of Health, Bethesda, MD; 21 Department of Pathology, University of Utah School of Medicine, Salt Lake City, UT; 22 Department of Diagnostic, Molecular, and Interventional Radiology, Icahn School of Medicine at Mount Sinai, New York, NY; 23 Electromyography Section, National Institute of Neurological Disorders and Stroke, National Institutes of Health, Bethesda, MD; 24 Undiagnosed Disease Program, Office of the Clinical Director, National Human Genome Research Institute, National Institutes of Health, Bethesda, MD; 25 Division of Neurogenetics, Center for Neuroscience and Behavioral Medicine, Children’s National Health System, Washington, DC; 26 Radiology & Imaging Sciences Department, Clinical Center, National Institutes of Health, Bethesda, MD; 27 Otolaryngology Branch, Audiology Unit, National Institute on Deafness and Other Communication Disorders, National Institutes of Health, Bethesda, MD; 28 Pediatrics and Developmental Neuroscience Branch, National Institute of Mental Health, National Institutes of Health, Bethesda, MD; 29 Office of the Clinical Director, National Institute of Mental Health, National Institutes of Health, Bethesda, MD; 30 Rehabilitation Medicine Department, Clinical Center, National Institutes of Health, Bethesda, MD; 31 Ophthalmic Genetics and Visual Function Branch, National Eye Institute, National Institutes of Health, Bethesda, MD; 32 Laboratory on Quantitative Medical Imaging, National Institute of Biomedical Imaging and Bioengineering, National Institutes of Health, Bethesda, MD; 33 Division of Neuroradiology, Department of Radiology, Boston Children's Hospital, Boston, MA; 34 Department of Radiology, Harvard Medical School, Boston, MA; 35 Department of Cell, Developmental, and Regenerative Biology, Icahn School of Medicine at Mount Sinai, New York, NY; 36 Department of Pediatrics, Icahn School of Medicine at Mount Sinai, New York, NY; 37 Department of Clinical Genomics, Mayo Clinic, Rochester, MN

**Keywords:** Congenital facial weakness, Cranial nerve, Exome sequencing, Genome sequencing, Moebius syndrome

## Abstract

**Purpose:**

To explore the phenotypic spectrum and genetic etiologies of Moebius Syndrome (MBS), a rare neurological disorder defined by congenital, nonprogressive facial weakness and limitations in ocular abduction.

**Methods:**

We applied strict diagnostic criteria and conducted clinical phenotyping of 149 individuals with MBS. Subsequently, we performed exome and/or genome sequencing on 67 of these individuals and 117 unaffected family members.

**Results:**

All 149 individuals had sporadic MBS, with no recurrence within or across generations. Common co-occurring phenotypes included tongue hypoplasia (81.9%), micrognathia (66.4%), congenital talipes equinovarus (42.3%), major limb anomalies (31.5%), intellectual disability (30.9%), sleep difficulties (22.8%), and Poland anomaly (14.1%). Filtering for rare de novo or autosomal recessive single-nucleotide, insertion/deletion, and structural variants in the sequenced cohort yielded 173 single-nucleotide variant/indels in 113 genes. Although we prioritized 7 candidate genes with de novo variants and 5 with biallelic variants, no compelling recurrently mutated genes were identified. Similarly, we found no convincing variants in 2 putative genes previously implicated in MBS: *PLXND1* (HGNC:9107) and *REV3L* (HGNC:9968).

**Conclusion:**

We did not identify a strong or unifying germline genetic etiology for MBS. Future studies may explore alternative causes, including environmental exposures, somatic variants, and/or complex inheritance patterns affecting brainstem and organ embryogenesis.

## Introduction

**Figure 1:**
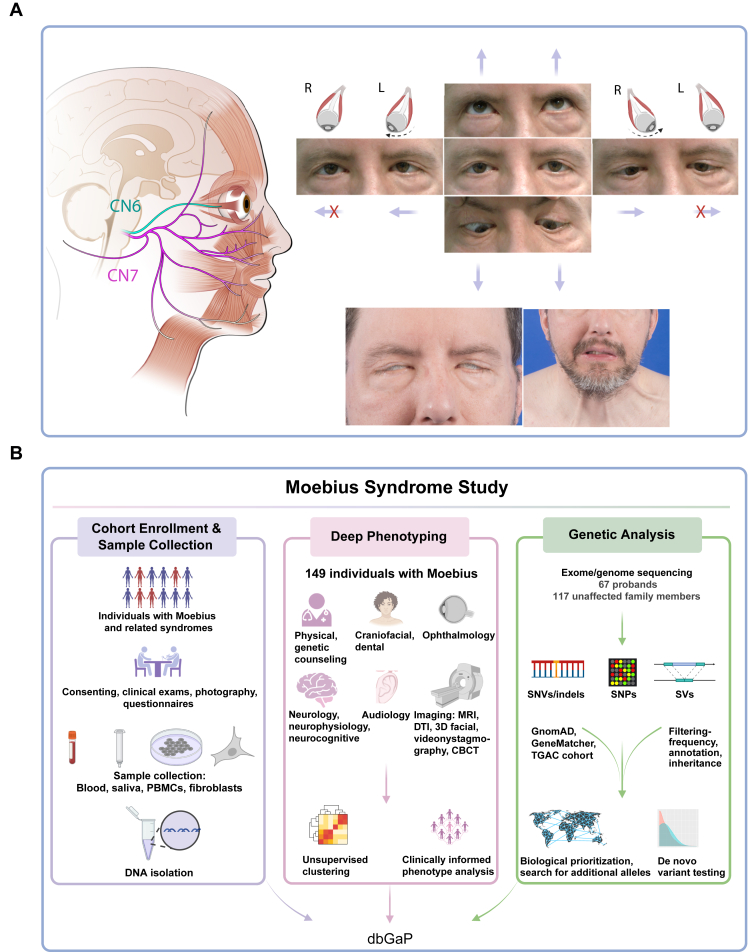
**MBS definition and study workflow.** A. Schematic of human right-sided CN6 (abducens nerve, green) and CN7 (facial nerve, purple) innervating the lateral rectus and muscles of facial expression, respectively (left). Photos of an individual with MBS highlighting bilateral restricted abduction with full vertical eye movements (right top) and bilateral facial weakness (right bottom). Note mask-like facies, lagophthalmos, asymmetry of nasolabial folds with attempted smile, and engagement of the platysma to increase facial movement. CN6, cranial nerve 6; CN7, cranial nerve 7; L, left; R, right; X, restricted or absent ocular motility. B. Graphical depiction of study enrollment and phenotype and genotype analyses workflow. CBCT, cone-beam computed tomography; dbGaP, the database of Genotypes and Phenotypes; DTI, diffusion tensor imaging; indel, small insertions and deletions less than 50 nucleotides in length; MBS, Moebius Syndrome; MRI, magnetic resonance imaging; PBMCs, peripheral blood mononuclear cells; SNP, single-nucleotide polymorphism; SNV, single-nucleotide variant; SV, structural variant; TGAC, The Genomic Ascertainment Cohort.

In 1888, Moebius syndrome (MBS; OMIM 157900) was first defined as congenital bilateral facial and abducens palsies without ptosis or limited vertical eye movements (Figure 1A).^1^ Subsequent investigations have further explored the clinical features and etiology of MBS. Most cases appear sporadic, and although heritability has occasionally been reported,^2^^,^^3^ those investigations included individuals with distinct “Moebius-like” phenotypes, such as hereditary congenital facial paresis,^4^^,^^5^ Duane retraction syndrome,^6^ congenital fibrosis of the extraocular muscles (CFEOM),^7^ facioscapulohumeral muscular dystrophy,^8^ and congenital myopathies associated with facial weakness and ophthalmoplegia, such as Carey-Fineman-Ziter syndrome (*MYMK* [HGNC:33778], OMIM 254940)^9^ or *STAC3* (HGNC:28423, OMIM 255995)- and *RYR1* (HGNC:10483, OMIM 255320)-related myopathies.^10^^,^^11^ In 2007, an international consortium aimed to distinguish MBS from related phenotypes by reaffirming MBS as congenital, nonprogressive unilateral, or bilateral facial weakness co-occurring with limited abduction of 1 or both eyes.^12^ Subsequently, the additional criteria of full vertical ocular motility without ptosis were emphasized,^13^ consistent with the original case reports in 1888, and over half of affected individuals were found to have deficits of both adduction and abduction.^13^, ^14^, ^15^ Typical brain magnetic resonance imaging (MRI) findings include markedly hypoplastic abducens nerve (cranial nerve [CN] 6, which innervates the lateral rectus muscle that abducts the eyes); variably hypoplastic facial nerve (CN7, which innervates the muscles of facial expression), and variably small lateral rectus and facial muscles. Beyond its core features, MBS may present with additional neurological abnormalities, such as intellectual disability (ID), autism, and lower CN dysfunction (CNs 9-12), as well as nonneurological phenotypes, including Poland anomaly, limb malformations, and/or other organ dysmorphogenesis.^6^^,^^16^

Human CNs that provide afferent and efferent innervation principally to the structures of the head and neck arise from distinct brainstem nuclei and develop between 4 to 8 weeks after fertilization. Abducens motor neurons are born in rhombomeres (r) 5 and 6, and their axons course ventrally from the abducens nucleus and exit at the junction of the pons and medulla to innervate the ipsilateral lateral rectus muscle that pulls the globe outward (abduction). Importantly, the abducens nucleus also contains a population of interneurons, whose axons decussate and ascend in the medial longitudinal fasciculus to the medial rectus subnucleus of the oculomotor (CN3) nucleus. These intermingled neurons in the abducens nucleus allow for simultaneous activation of the ipsilateral lateral rectus and contralateral medial rectus muscles, thus creating the anatomic substrate for conjugate horizontal gaze.^17^ Facial branchiomotor neurons (CN7) originate in r4 and migrate caudally to r6; their axons trail behind, wrapping around the abducens nucleus and exiting the pons at r4 to innervate the ipsilateral muscles of facial expression.^18^

The etiology of MBS is unknown. However, genetic and environmental causes have been postulated, including focal hypoperfusion secondary to vascular disruption.^19^ Various prenatal exposures, including misoprostol, thalidomide, ergotamine, cocaine, and maternal homocystinuria, have been associated with an increased risk for MBS.^20^, ^21^, ^22^, ^23^, ^24^ Rare individuals have been reported to harbor heterozygous de novo variants in *PLXND1* (HGNC:9107, OMIM 604282), *REV3L* (HGNC:9968, OMIM 602776),^25^^,^^26^ and *CHN1* (HGNC:1943, OMIM 118423),^27^ as well as complex rearrangements disrupting semaphorin-plexin genes.^28^ Most of these individuals, however, did not meet MBS diagnostic criteria, and additional reports have not substantiated MBS heritability,^7^^,^^29^^,^^30^ supporting postzygotic (somatic) genetic or nongenetic etiologies.

In this study, we assembled a large cohort of individuals who met diagnostic criteria for MBS and performed additional clinical phenotyping and, in a subset, exome sequencing (ES) and/or genome sequencing (GS) to search for possible genetic etiologies of MBS.

## Materials and Methods

Additional details on participant recruitment, experimental procedures, data collection, and analysis are provided in Supplemental Information.

### Participant enrollment and phenotyping

A collaboration between the National Institutes of Health (NIH), Icahn School of Medicine at Mount Sinai, and Boston Children’s Hospital was established to investigate MBS and related syndromes. Clinical protocols (ClinicalTrials.gov ID: NCT02055248, NCT03059420) and signed reliance agreements were implemented to facilitate the sharing of identifiable participant information. Inclusion criteria for this study were congenital, nonprogressive facial weakness and limited abduction of 1 or both eyes. Participants were excluded if they had restriction of supraduction above the −3 position preoperatively, or any limitation of supraduction in combination with ptosis. Other congenital anomalies could be present or absent. To standardize data collection, 27 tools including participant questionnaires and physician physical exam forms were developed, and data were stored within a Research Electronic Data Capture (REDCap) registry. We enrolled 203 individuals referred with MBS or related phenotypes and, after a comprehensive review of ophthalmology, neurology, and clinical genetics records, retained 149 individuals who met MBS diagnostic criteria (Figure 1B). The 54 excluded individuals were referred to the study with the diagnosis of MBS and enrolled but on phenotype review were found to have moderate to severe uni- or bilateral vertical gaze palsy with ptosis (including some diagnosed with CFEOM), isolated congenital facial palsy (CFP), or were diagnosed with congenital myopathies after electrophysiology and molecular genetics testing. Ptosis was defined as weakness of the levator palpebrae superioris muscle that could be attributed to dysfunction of the oculomotor nerve (CN3) and did not include palpebral fissure dysmorphology.

One hundred and fifteen of the 149 participants were enrolled at Moebius Syndrome Foundation family conferences, during which they underwent comprehensive evaluations by a clinical geneticist, ophthalmologist, and neurologist. Additionally, photos, videos, and saliva and/or blood specimens were collected. A subset of affected individuals (*n* = 31) and unaffected family members (*n* = 50) underwent 3- to 5-day multidisciplinary evaluations by genetics, ophthalmology, neurology, audiology, neuropsychology, dental/craniofacial, and physiatry consultants at the NIH Clinical Research Center (Supplemental Phenotyping Methods). When possible, testing included structural brain MRI, diffusion tensor imaging, and electrophysiological studies, some previously reported.^31^^,^^32^ Phenotype data were curated and reviewed for accuracy by 2 clinical geneticists and a genetic counselor and discussed with study team members at monthly group project meetings. Frequencies of co-symptomatology are presented as percent of individuals with the respective phenotype out of the total cohort and out of the number of individuals with available data (Supplemental Table 1). Phenotype frequencies in the full cohort were compared with the sequenced cohort and to cohorts previously reported in the literature (Supplemental Tables 2 and 3), whereas available brain imaging data were reviewed by 2 neuroradiologists (Supplemental Table 4).

The pheatmap R package was used to cluster affected individuals based on the presence or absence of 6 associated features (congenital talipes equinovarus [clubfoot], major limb anomalies, ID and/or autism spectrum disorders, hearing loss, cleft palate or bifid uvula, and Poland anomaly; listed from highest to lowest frequency).

### GS and ES

#### Sequencing, alignment, and variant calling

Exome sequences (ES) or genome sequences (GS) were generated at the National Human Genome Research Institute, NIH Intramural Sequencing Center or by the NIH Gabriella Miller Kids First Pediatric Research Program from salivary or blood-sourced DNA for 67 probands, including 26 of 31 probands evaluated at the NIH Clinical Research Center, and 117 unaffected family members (ES = 63; GS = 54). These included 52 trios, defined as the MBS proband and the 2 unaffected biological parents. ES and GS methods were described previously.^9^^,^^33^ Paired-end sequence reads were mapped to the human reference genome GRCh37 using “bwa mem” (v0.7.17)^34^ and germline single-nucleotide variants (SNVs) and insertion/deletions (indels) were called following the GATK best practice variant calling guidelines. A structural variant (SV) calling pipeline was developed and applied to GS data. This pipeline called SVs using 11 callers, merged different call sets into consensus SV sites, and completed joint SV genotyping across all individuals (Supplemental Genotyping Methods).

#### Quality control of genotype calls

To confirm genotype quality, we assessed the concordance of SNV/indel genotypes called by either GATK or Octopus (v0.6.3-beta)^35^ using bwa alignment of the bam files to assess concordance. We also compared genotype calls for samples sequenced with both ES and GS (Supplemental Genotyping Methods). We used genotypes prepared with the GATK best practice pipeline for all downstream analyses.

#### Filtering SNV/indel lists for candidate variants

Variant calls for the 52 trios and 3 quads were filtered with Qiagen QCI Interpret based on confidence, population frequency, mode of inheritance, and predicted deleteriousness using CADD v1.6 PHRED, pLI, LOEUF, missense *z*-scores, and AlphaMissense scores^36^, ^37^, ^38^, ^39^ (Supplemental Genotyping Methods). Candidate genes were further prioritized based on expression patterns in CN6 and CN7 in *Isl+* developing rhombomere 4 motor neurons of embryonic mice,^5^^,^^40^ phenotypes in animal models, phenotypes in humans including neurological/neurodevelopmental phenotypes, cataloged with biological terms, such as “facial weakness” and “neurological abnormality,” and based on interactions or associations with known facial weakness disease-causing genes. To identify additional alleles, prioritized novel candidate genes and genes with less established human phenotype associations were submitted to Matchmaker exchange.^41^^,^^42^ Twelve additional probands with 0 or 1 parent (ie, nontrios) were sequenced, and data analysis for this group was limited to variants in candidate genes in the literature or those identified from the trio data in this study. Mothers of MBS probands were screened for variants with minor-allele frequency <0.5% in 79 thrombophilia and bleeding disorder genes.

#### Filtering SVs lists for candidate variants

SVs were filtered for de novo and homozygous recessive inheritance models and by allele frequencies determined using publicly available GS data that had been genotyped using the same calling pipeline (Supplemental Genotyping Methods).

#### De novo variant rate analysis

To determine whether individuals with MBS have increased rare coding/splice site de novo SNVs (DNVs), 28 ES and 27 GS from the 55 MBS trios/quads were analyzed alongside published DNVs from 1449 trio GS from the TOPMed Program^43^ and from 1789 control trio ES from Simons Simplex Consortium (SSC).^44^ Numbers of DNVs per individual were counted, and DNV rates in MBS cohort versus each control population were compared by Mann-Whitney 2-sided U test. To control for different sequencing or processing pipelines, GS of 29 MBS trios were also compared with 40 trios consisting of individuals with non-MBS congenital cranial dysinnervation disorders or their unaffected siblings and their parents who were sequenced in parallel and called jointly with the MBS trios (dbGaP accession number phs001247.v1.p1; sequencing and processing details summarized previously).^33^ Number of DNVs per individual and paternal ages at conception were obtained. DNV rates in the 2 populations were compared by using Mann-Whitney 2-sided U test and Poisson regression with paternal age at conception as covariates (Supplemental Methods and Results).

## Results

### MBS demographic and phenotypic evaluations

**Figure 2:**
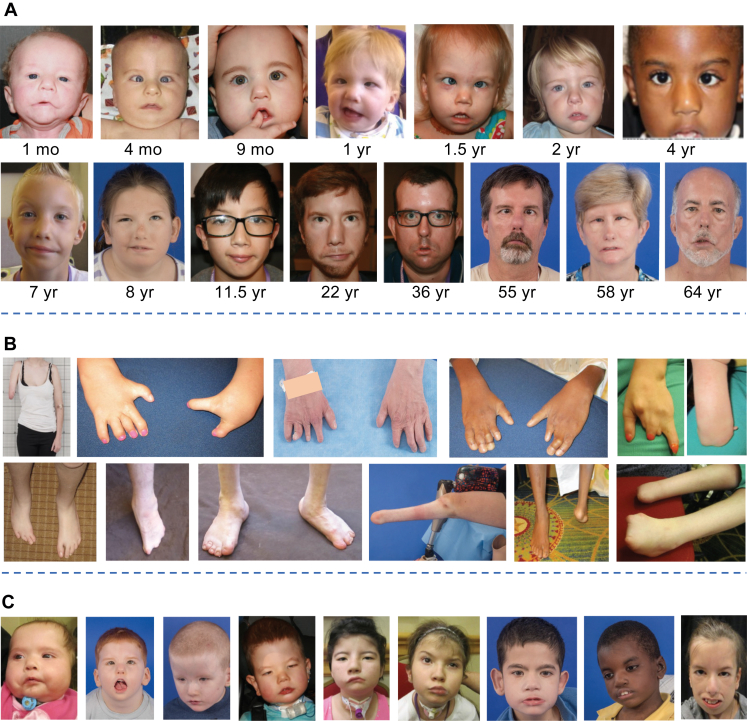
**MBS clinical phenotypes.** A. Representative facial images for both sexes across the lifespan reveal a predominant phenotype of asymmetric smiles, esotropia, and a spectrum of dysmorphic features including low-set ears, short nose, midface hypoplasia, underdeveloped nasolabial or forehead creases, narrow mouth with downturned corners, and micrognathia. B. Representative upper and lower limb anomalies, including transverse limb reduction defect/absent forearm and hand, brachydactyly, brachysyndactyly, absent digit(s), ectrodactyly, absent foot or toes, metatarsus adductus, and clubfoot. C. Severe phenotypes include lower cranial nerve anomalies, such as tongue hypoplasia/furrowed tongue, tracheostomy and/or gastrostomy tube for feeding, mild to moderate intellectual disability, and/or autism spectrum disorder. MBS, Moebius Syndrome.

**Figure 3:**
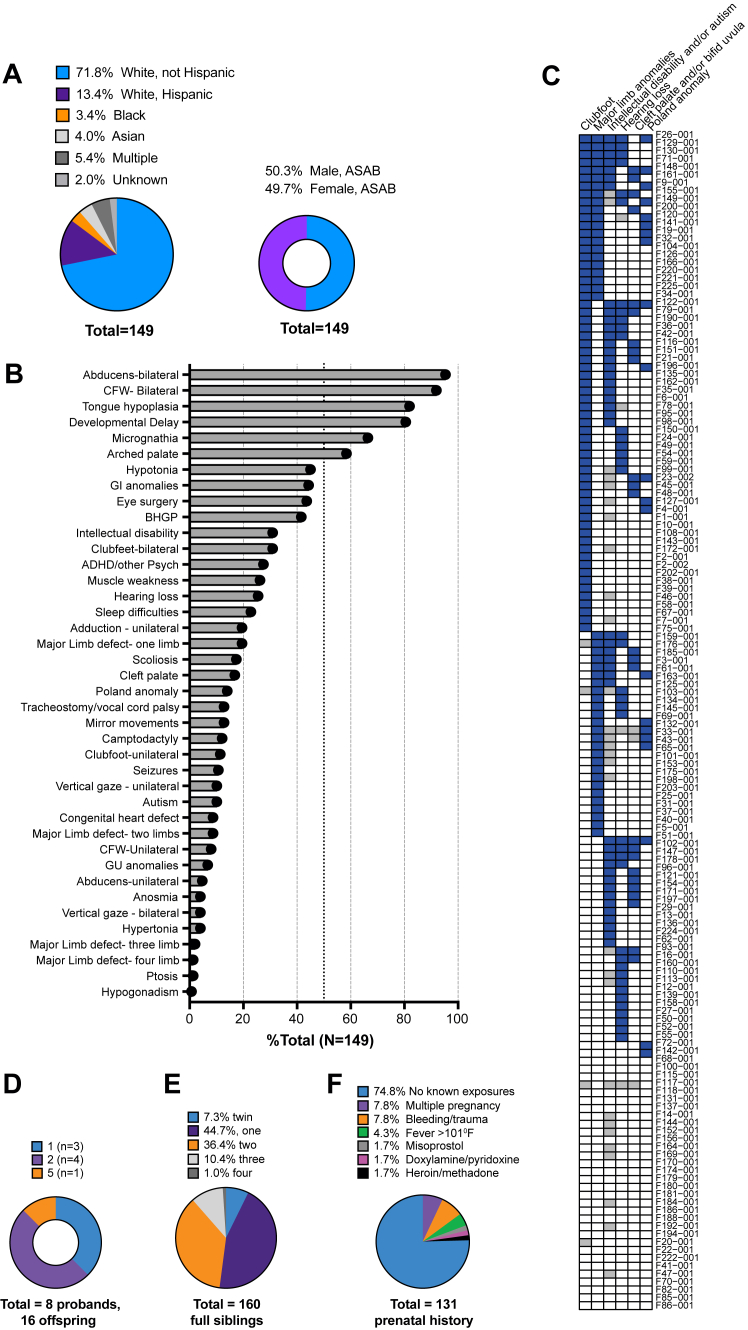
**Demographics and phenotypic spectrum of MBS cohort.** A. Self-reported race, ethnicity, and sex distribution in the study cohort are presented in pie charts with slice sizes proportional to the respective frequencies. ASAB, assigned sex at birth. The study represents a large and diverse cohort of MBS individuals, with equal male/female distribution. B. Percent incidence of key phenotypic features in the cohort (*n* = 149) sorted by frequency. ADHD, attention-deficit hyperactivity disorder; BHGP, bilateral horizontal gaze paresis; CFW, congenital facial weakness; GI, gastrointestinal; GU, genitourinary; trach, tracheostomy. C. Clustering of participants given presence of 6 features (clubfoot, major limb anomalies, intellectual disability and/or autism, hearing loss, cleft palate or bifid uvula, and Poland anomaly), with features listed from highest to lowest frequency for the entire MBS cohort (*N* = 149). To visualize, probands with the most frequent characteristic were identified and sorted based on frequency of the remaining 6 characteristics in descending order, followed by the next highest frequent characteristic. Phenotype data are visualized using the pheatmap R package. Each column is a feature and each row an MBS proband, denoted by their code number. Filled blue boxes denote the presence of that phenotype feature in the proband, whereas filled gray boxes denote absence of available data for that feature in that proband. D. Offspring number per MBS proband are depicted. A total of 8 MBS individuals had 1 to 5 children each for a total of 16 offspring, all unaffected. E. The total number of siblings of MBS probands. A total of 160 full siblings were reported in the family pedigrees of MBS individuals in the cohort, all unaffected. Six individuals had a living unaffected fraternal twin, and 3 had a prenatal history positive for a vanishing fetus. F. Prenatal histories and environmental exposures during pregnancy by self-report. MBS, Moebius Syndrome.

After the multidisciplinary team assessment, 149 individuals were confirmed to have MBS (age range 1 month to 74.6 years, mean ± SD: 18.8 ± 16.9, median 13.0 years) (Figure 1, Figure 2A-C, Supplemental Figure 1A and B); breakdown of self-identified race, ethnicities, and sex assigned at birth are provided (Figure 3A). Key clinical features are presented by frequency in descending order in the full phenotyped cohort (*n* = 149) (Figure 3B), as well as in the subgroup without prior eye surgeries (Supplemental Figure 2A and B). All probands had congenitally limited ocular abduction and facial palsy, which were bilateral in 95.3% and 92.0%, respectively. Bilateral or unilateral restricted ocular adduction was present in 46.3% and 19.5%, respectively. Mild limitations in unilateral or bilateral vertical ocular motility below the −3 position were noted in 10.1% (*n* = 15) and 5.3% (*n* = 8), respectively, and none had ptosis. In total, 43.6% of the entire cohort was assessed after eye muscle surgery, including all, but 1 (with mild bilateral restriction in supraduction) of these 23 individuals. We compared frequencies of each phenotypic feature between individuals with and without eye surgeries. No statistically significant differences were found, except for a borderline higher prevalence of bilateral adduction deficit in individuals who had undergone surgery (Supplemental Table 5A).

Co-occurring neurological phenotypes included ID, defined as significant impairments in both intellectual functioning and adaptive behavior (30.9%), attention-deficit/hyperactivity disorder or other psychiatric diagnoses, such as anxiety and/or depression (27.5%), autism spectrum disorders (10.1%), mirror movements (12.8%), and/or seizures (10.7%). Sensorineural and/or conductive hearing loss was identified in 26.2%. Common craniofacial features included tongue hypoplasia (81.9%), micrognathia (66.4%), abnormal palate (58.4%), and cleft palate/bifid uvula (16.8%), whereas a tracheostomy was required in 12.8%. The incidence of congenital heart defects was 8.7%. Poland anomaly, identified in 14.1%, was defined as pectoralis muscle hypoplasia with hypoplastic or displaced nipple with or without variable degree of ipsilateral hand and digit anomalies, including brachymelia, absent/small hand and/or oligodactyly, brachy and/or symbrachydactyly, thumb hypoplasia, and/or camptodactyly. Gastrostomy tube was required by 19.5%, and gastroschisis was present in 2.0%. Scoliosis was present in 17.4%. Limb involvement was common (Figure 2B): 42.3% had clubfoot (talipes equinovarus), of which 73.0% were bilateral, whereas 31.5% had major limb anomalies defined as limb reduction, complete syndactyly, missing digits, and other complex limb differences. Of these, involvement of 1 (61.7%) or 2 (27.7%) limbs was more common than 3 (8.5%) or 4 (4.3%) (Figure 2B, Supplemental Figure 1, Supplemental Table 1).

A comparison of frequencies of co-occurring phenotypes revealed several notable associations (Supplemental Table 5B). Both ID and seizures were significantly associated with gastrointestinal anomalies, vocal cord paralysis/tracheostomy, and cleft palate. Major limb anomalies were positively associated with high-arched palate but not with other organ dysmorphogenesis, ID, or neurological symptoms. When individuals with prior eye surgeries were excluded, the Pearson χ^2^ *P* values changed; however, the significant associations of these co-occurring phenotypes remained (Supplemental Table 5C). Pectoralis hypoplasia (Poland anomaly) was associated with limb defects but not with the other phenotypic features. Additionally, scoliosis (27% vs 9.7%, χ^2^ *P* = .009) and micrognathia (79% vs 55%, χ^2^ *P* = .003) were more frequently observed in female participants.

To assess patterns of co-occurring features, we visualized the presence or absence of 6 features (clubfoot, major limb anomalies, ID and/or autism, hearing loss, cleft palate and/or bifid uvula, and Poland anomaly) for each individual in the entire cohort (Figure 3C) and in the subgroup without prior eye surgeries (Supplemental Figure 2A and B). No distinct phenotypic subclusters emerged from this analysis.

### Family history and prenatal findings

Among 140 probands with family history available, all were sporadic/simplex cases with no known horizontal or vertical transmission of the condition (Figure 3D and E). There were 160 full siblings across the cohort, none of whom had MBS. Eight probands had a total of 16 offspring (1-5 offspring per proband), and again, none had MBS. Ten cases were the product of a recognized multiple gestation (Supplemental Table 1). Additionally, bleeding or trauma during early gestation (8-12 weeks) was reported in 9 singleton pregnancies. Among 131 probands with available prenatal history, exposures to misoprostol, heroin/methadone, or doxylamine/pyridoxine were each reported twice (6 pregnancies total, Figure 3F).

### Brain imaging findings

**Figure 4:**
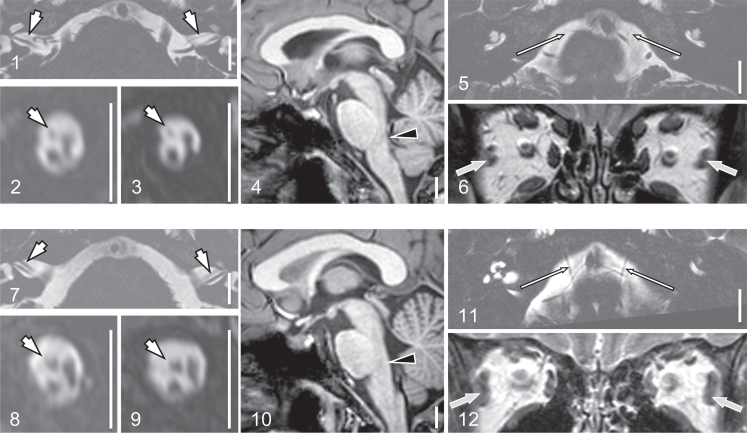
**Brain magnetic resonance imaging findings in MBS.** Features of MBS on brain MRI from a 33-year-old female proband with MBS and clubfoot (F58-001, panels 1-6) compared with a 24-year-old individual with hemifacial myohyperplasia in the absence of MBS and intracranial abnormalities (panels 7-12). CN7 (short white arrows in 1-3 and 7-9) is hypoplastic in MBS (1-3) but is well seen in the control (7-9). The pontine tegmentum (black arrowhead in 4) is hypoplastic in F58-001, resulting in a concavity in the dorsal brainstem. CN6 cannot be identified in the MBS proband (long white arrows in 5) but is well seen traversing the prepontine cistern in the control (long white arrows in 11). The lateral rectus muscles are relatively small (gray arrows in 6) in MBS compared with control (gray arrows in 12). Imaging sequences: T2 weighted volume isotropic turbo spin echo images reformatted axial through the internal auditory canals (1,7) and oblique axial through the prepontine cisterns (5,11); sagittal reformats of 0.5 mm isotropic balanced fast field echo through the right (2,8) and left (3,9) IACs at sites of arrows in panels 1 and 7; midline sagittal image from 1mm isotropic 3D (4,10); coronal fast spin echo T2 through the orbits (6,12). Scale bars, 1 cm (white bars in 1-5 and 7-11). See also Supplemental Figure 3. MBS, Moebius Syndrome.

Brain MRIs from 15 probands scanned at the NIH were reviewed (Figure 4, Supplemental Figure 3A-D, Supplemental Table 4). All had absent or hypoplastic CN7. Ten had absent or hypoplastic CN6, whereas the remaining 5 individuals had nondiagnostic imaging in which abnormalities in CN6 could not be determined. Four of the 10 individuals with abnormalities in visualization of CN6 had small lateral rectus muscles. Two of the 5 individuals with nondiagnostic imaging for CN6 had small lateral rectus muscles. Additional findings included unilateral hypoplastic CN1 or CN12 each in 1 proband. None of the 15 individuals imaged had visible CN3, CN9, CN10, or CN11 anomalies or small superior, medial, or inferior rectus muscles. A small anterior commissure was present in 5 probands and prominent ventricles in 2 probands.

### MBS genetic analyses

Of 149 MBS probands, 34 underwent ES only (3 quads, 19 trios, 4 duos, and 8 singletons), 6 underwent ES and GS (F4-001, F21-001, F36-001, F78-001, F79-001, and F82-001; all trios), and 27 underwent GS only (all trios). ES and GS data had high quality and concordance of called SNVs (Supplemental Table 6), and ES were used for all downstream analyses of the 6 probands with both ES and GS, except for the de novo variant rate analysis comparing GS of Gabriella Miller Kids First-sequenced individuals. Ancestries were determined based on genomic data and included 2 African, 3 Asian, 49 European, 12 Hispanic, and 1 individual with multiple ancestries; genetically inferred and self-reported ancestries were concordant in 61 of 67 individuals (91.0%) (Supplemental Genotyping Methods, Supplemental Figure 4, Supplemental Table 7).

#### Prioritized genetic variants

Among the 67 probands with sequence data, variant filtering yielded 173 SNV/indels in 113 genes (Supplemental Table 8); 172 of 173 variants (99.4%) were called by both GATK and Octopus callers. Among 27 trios with GS, SV filtering yielded 46 variants in 23 trios (Supplementary Genotyping Methods, Supplemental Table 9). We identified no definitive causal variants in genes shared by multiple MBS individuals but flagged several candidate variants of uncertain significance, highlighted below.

Two genes were mutated in more than 1 proband: *TTN* (HGNC:12403) (F49-001, F82-001, F151-001, F222-001, and F224-001) and *COL24A1* (HGNC:20821) (F42-001 and F220-001). Both genes harbored rare compound heterozygous missense variants. *TTN* and *COL24A1* are the #1 and #1776 largest genes in the human genome, respectively, and thus are large targets for variation by chance. Both also have low human missense constraint (missense *z* = −1.1 and −0.21, respectively). *TTN* variants are associated with myopathies, muscular dystrophies, and cardiomyopathies (OMIM 188840), which are inconsistent with the phenotypes of these probands, and *COL24A1* variants have not been associated with Mendelian phenotypes. Moreover, mouse *Col24a1* and *Ttn* are not expressed by developing CN6/CN7 motor neurons at E9.5-E12.5.^40^ Thus, both genes were deprioritized as MBS candidates.

Of the 113 candidate genes that were retained after filtering of variants based on confidence, population frequency, mode of inheritance, and computationally predicted deleteriousness scores, 12 were prioritized based on (1) gene expression in *Isl1*^+^ CN6 and CN7 developing rhombomere 4 motor neurons in embryonic mice,^5^^,^^40^ (2) known phenotypes in animal models and humans, and/or (3) relations to known congenital facial weakness disease-causing genes. This curated selection included 7 genes harboring de novo variants (*USP15* [HGNC:12613], *STMN3* [HGNC:15926], *PALM* [HGNC:8594], *CNTRL* [HGNC:1858], *KPNA3* [HGNC:6396], *POU2F1* [HGNC:9212]*, MINDY1* [HGNC:25648])*,* and five genes with biallelic variants (*ZNF407* [HGNC:19904]***,*** *PBXIP1* [HGNC:21199]*, KCNAB2* [HGNC:6229]*, ZRANB1* [HGNC:18224]*,* and *MORC2* [HGNC:23573]) (Table 1). The 12 candidate genes were identified in only 9 probands because F45-001 had variants in 2 (*CNTRL* and *ZNF407*), and F75-001 had variants in 3 candidates (*KPNA3*, *ZRANB*, and *PBXIP1*). Eleven of the genes (all except *KCNAB2*) are expressed in developing facial motor neurons in mice. Although variants in 8 (*USP15*, *PALM*, *CNTRL*, *ZNF407*, *PBXIP1*, *ZRANB1*, *POU2F1*, and *MINDY1*) have been implicated in neurodevelopmental phenotypes and variants in 3 (*KCNAB2*, *KPNA3*, and *MORC2*) in neurological phenotypes (Supplemental Table 10), none of the reported individuals harboring these variants met criteria for MBS. *STMN3* and *PBXIP1* were also prioritized because of similarities or functional associations with the congenital facial weakness genes *TUBB3* (HGNC:20772) and *HOXB1* (HGNC:5111), respectively. Finally, 3 of these genes encode deubiquitinating enzymes (*USP15*, *MINDY1*, and *ZRANB1*), whereas *KPNA3* facilitates the nuclear import of the deubiquitinase *ATXN3*.

**Table 1:** Prioritized genetic variants in individuals with Moebius syndrome

ID	Gene	HGNC:Gene_ID	Variant Genomic Location	Variant (Transcript, CDNA, Protein)	Inheritance	Frequency	CADD, Alpha Missense Scores	ACMG/AMP/ClinGen Classification Criteria	Expressed in CN6/CN7^a^	Diagnoses in Addition to MBS
F21-001	*USP15*	HGNC:8594	chr12 NC_000012.11:g.62798141A>G	NM_001252078.2:c.2932A>G, p.(Met978Val)	De novo	0	23.1, 0.094	VUS (PM2)	Yes/Yes	Clubfoot, cleft palate, ASD, autism, DD, G-tube, short stature
F23-002	*STMN3*	HGNC:23573	chr20 NC_000020.10:g.62275566C>T	NM_015894.4:c.115+1G>A	De novo	0	34, NA	VUS (PM2, PP3)	Yes/Yes	Clubfoot, Poland anomaly, bifid uvula
F26-001	*PALM*	HGNC:25648	chr19 NC_000019.9:g.746683G>A	NM_002579.3:c.1033G>A, p.(Gly345Ser)	De novo	0	25.2, 0.212	VUS (PM2)	Yes/Yes	Clubfoot, Poland anomaly, brachysyndactyly, scoliosis
F36-001	*KCNAB2*	HGNC:19904	chr1p36.31 (6095834-6108518)x0	NA	AR	0	NA, NA	NA	No/No	Clubfoot, seizures, mild ID, hearing loss, low set ears, high palate
F45-001	*CNTRL*	HGNC:1858	chr9 NC_000009.11:g.123850681C>T	NM_007018.6:c.77C>T, p.(Ser26Phe)	De novo	0	24, 0.114	VUS (PM2)	Yes, low/Yes	Clubfoot, seizures, hypoglossia
F45-001	*ZNF407*	HGNC:19904	chr18 NC_000018.9:g.72345298A>G and g.72345374A>G	NM_017757.3:c.2323A>G, p.(Ile775Val) and c.2399A>G, p.(His800Arg)	AR	0; 1.2e-3	22.7, 0.228 15.78, 0.089	VUS (PM2); VUS (PM2)	Yes, low/Yes, low	See above
F75-001	*KPNA3*	HGNC:6396	chr13 NC_000013.10:g.50279798T>C	NM_002267.4:c.1331A>G, p.(Asp444Gly)	De novo	0	23.5, 0.125	VUS (PM2)	Yes/Yes	Micrognathia, hypoglossia, clubfeet, DD
F75-001	*PBXIP1*	HGNC:21199	chr1 NC_000001.10:g.154920759G>A and g.154918403G>A	NM_001317734.2:c.406C>T, p.(Arg136Trp); and c.1660C>T, p.(Arg554Trp)	AR	2.6e-5; 1.5e-4	23.6, 0.104 26.7, 0.12	VUS (PM2); VUS (PM2)	No/Yes, low	See above
F75-001	*ZRANB1*	HGNC:21199	chr10 NC_000010.10:g.126655187C>A and g.126655329G>A	NM_017580.3:c.839C>A, p.(Ala280Asp); c.981G>A, p.(Met327Ile)	AR	0; 0	28.8, 0.871 22.3, 0.671	VUS (PM2, PP3); VUS (PM2)	Yes, low/Yes, low	See above
F79-001	*MORC2*	HGNC:18224	chr22 NC_000022.10:g.31324008C>G and g.31331293C>T	NM_014941.3:c.2842G>C, p.(Glu948Gln); c.1568G>A, p.(Arg523His)	AR	3.2e-5; 1.8e-4	27.9, 0.839 25.6, 0.142	VUS (PM2); VUS (PM2)	Yes, low/Yes, low	Clubfoot, posterior cleft palate, G-tube, hypothyroidism, autism, severe ID
F121-001	*POU2F1*	HGNC:23573	chr1 NC_000001.10:g.167381322C>A	NM_002697.4:c.1682C>A, p.(Ser561Tyr)	De novo	0	27.1, 0.158	VUS (PM2)	Yes, low/Yes, low	Cleft palate, kyphosis, sleep apnea, ID
F137-001	*MINDY1*	HGNC:18224	chr1 NC_000001.10:g.150974825G>A	NM_001163258.3:c.269C>T, p.(Thr90Ile)	De novo	0	23.3, 0.114	VUS (PM2)	No/Yes, low	Camptodactyly, narcolepsy, MM

Variant genomic locations are provided using GRCh37/hg19 coordinates. Allele frequencies were derived from gnomAD v3 for SNVs/indels, or from gnomAD SV v2.1 for SVs.

*ASD*, atrial septal defect; *CNTRL*, centriolin; *DD*, developmental delay; *G-tube*, gastrostomy tube; *ID*, intellectual disability; *KCNAB2*, potassium channel, voltage-gated, shaker-related subfamily, beta member 2; *KPNA3*, karyopherin subunit alpha 3; *LB*, likely benign; *LP*, likely pathogenic; *MBS*, Moebius syndrome; *MINDY1*, MINDY lysine 48 deubiquitinase 1; *MM*, mirror movements; *MORC2*, Morc family CW-type zinc-finger protein 2; *PALM*, paralemmin; *PBXIP1*, PBX-homeobox interacting protein 1; *POU2F1*, POU class 2 homeobox 1; *STMN3*, stathmin 3; *USP15*, ubiquitin-specific peptidase 15; *ZNF407*, zinc-finger protein 407; *ZRANB1*, zinc-finger RANBP2-type containing 1.

a Embryonic mouse CN6/CN7 expression data were derived from published RNA sequencing data of wild-type E9.5-E12.5 mice (Tenney et al^40^).

Additional details regarding the prioritized candidate genes and overlapping non-MBS phenotypes with the MBS individuals in this study are available in Supplemental Information, Supplemental Table 10, and Supplemental Results. These 12 genes were submitted to Matchmaker exchange and to the reverse phenotyping core at National Human Genome Research Institute, NIH (https://rpc.nhgri.nih.gov),^45^ but matches did not identify individuals with MBS or related CFP or ocular phenotypes.

#### Other genetic observations

Variants were also considered in *PIEZO2* (HGNC:26270) NM_022068.4:c.1535G>A, p.(Ser512Asn) seen in F32-001, *CHRNE* (HGNC:1966) NM_000080.4:c.103T>C, p.(Tyr35His) in F37-001, and *MEPE* (HGNC:13361) NM_001184697.2:c.278del, p.(Ser93IlefsTer3) in F5-001 because these 3 disease-associated genes, each of which can exhibit dominant inheritance, may cause symptomatology that could be mistaken for MBS including ophthalmoplegia (*PIEZO2*), myasthenia leading to CFP (*CHRNE*), and hereditary CFP with otosclerosis (*MEPE*), respectively. We compared the phenotype of each proband with the known disease phenotype and do not believe that these variants are causative in our participants (Supplemental Results, Supplemental Table 11). Finally, it has been reported that DNVs in *PLXND1* (HGNC:9107) and *REV3L* (HGNC:9968) cause MBS.^25^^,^^26^ We did not identify rare *PLXND1* or *REV3L* variants in our cohort.

#### De novo variant analysis

DNV rates differed significantly between MBS versus TOPMed but not between MBS versus SSC control cohorts (Supplemental Results). Because rates also differed significantly between TOPMed versus SSC control cohorts, we suspected that the former difference could be attributed to factors unrelated to MBS status, such as sequencing or variant calling methodologies. Thus, to control for differences in sequencing and processing methods, DNV rates were compared between probands with MBS and unaffected individuals included in a non-MBS cranial dysinnervation disorder cohort sequenced in parallel and called jointly. With these controls, there was no significant difference in DNV rates in MBS versus non-MBS cohorts (Supplemental Methods and Results, Supplemental Figure 5), supporting a consistent de novo variant rate across the various groups.

#### Variants in maternal thrombophilia genes

Analysis of sequencing data generated from 52 mothers of probands with MBS in 79 thrombophilia and bleeding disorder genes identified 9 SNVs of interest in 8 mothers (Supplemental Tables 12 and 13); however, these mothers did not exhibit symptoms of thrombophilia or bleeding diathesis; thus, their contribution to offspring with MBS is unlikely.

## Discussion

We present the results of a decade-long collaborative effort by a multidisciplinary team of researchers and clinicians from 3 institutions and families associated with the Moebius Syndrome Foundation to compile, to our knowledge, the largest and most diverse registry of individuals with MBS to date (*N* = 149, with 67 probands undergoing ES and/or GS). This study is further strengthened by strict criteria to exclude those with “Moebius-like” presentations included in previous genetic studies. Extensive phenotypic data, family histories and environmental exposures were collected in a systematic way, along with high quality ES and GS data, which are accessible to the broader genetics community via dbGaP for further exploration.

We applied stringent criteria for a MBS diagnosis, requiring both congenital abduction deficit and facial weakness and excluding preoperative restriction of supraduction above the −3 position or any limitation of supraduction in combination with ptosis, as well as individuals with other non-MBS diagnoses. We advocate for the universal adoption of these diagnostic parameters in future studies to facilitate accurate comparisons. An important distinction in the evaluation of individuals with MBS is that ptosis should be assessed as a weakness of the levator palpebrae superioris muscle and not due to dysmorphology causing palpebral fissure narrowing. In support of this, a previous study recommended that MBS not be considered as a possible diagnosis in individuals with any degree of vertical gaze limitation to avoid confusion with genetically defined conditions resulting from oculomotor nerve maldevelopment (CN3), including CFEOM in general and the *TUBB3* (HGNC:20772) E410K and R262H syndromes in particular.^13^^,^^46^ MRI and clinical examinations can facilitate this distinction. In this study, limited supraduction was observed in some individuals but was mild and did not co-occur with ptosis. Therefore, although mild supraduction limitation in the absence of ptosis is not a reason for exclusion, more severe restriction of supraduction or any limitation in combination with ptosis should motivate a search for causes of horizontal gaze limitation and facial palsy other than MBS.

A majority of our cohort (73.8%) was recruited from the Moebius Syndrome Foundation family conferences. These conferences attract individuals with MBS across a wide developmental and clinical spectrum, including children with more complex medical needs. Therefore, we believe that our cohort is not skewed toward either more or less severely affected individuals with MBS. Phenotypic data for many participants were collected through self-reported questionnaires, which are inherently subjective and prone to recall bias. To mitigate this limitation, however, we cross-referenced questionnaire responses with available medical records. In addition, photographs for dysmorphology exams and videos for eye movement evaluation were collected in a standardized way. Moreover, a subset of probands underwent detailed multisystem evaluations over 3 to 5 days at the NIH Clinical Research Center, including age-appropriate neurocognitive testing, brain MRI, diffusion tensor imaging, electrophysiology, audiology, and videonystagmography, among other studies. Notably, 91% of our ES (46% of entire ES/GS) cohort received this deep phenotyping, strengthening the validity of any possible genotype-phenotype associations drawn from our data.

An additional limitation of our study is that 43.6% of probands had undergone 1 or more eye surgeries. In MBS, individuals exhibit various degrees of esotropia, and strabismus surgery, if done, is typically done early in life to improve eye contact and restore binocular vision. It is likely that the patients who had surgeries presented with more severe strabismus, but we have no reason to believe that the severity of strabismus would have an association with extraocular findings. Indeed, we compared phenotype frequency distributions and association between phenotypes in individuals with and without eye surgeries and found no statistically significant differences, other than borderline higher prevalence of bilateral adduction deficit in individuals examined postoperatively, but that feature was not used for participant exclusion. (Supplemental Figure 2A and B, Supplemental Table 5A-C). This supports the robustness of our phenotypic findings and suggests that prior surgeries did not systematically bias our results. Nevertheless, we acknowledge that postoperative evaluations may limit our ability to detect certain mild congenital ocular motility abnormalities.

Our comprehensive phenotypic analyses enabled us to compare the frequencies of co-occurring phenotypes in our study relatively with other studies of MBS in the literature (Supplemental Table 3). We identified frequencies of 30.9% for ID and 10.1% for autism spectrum disorders, which fall within the broad ranges previously reported in smaller cohorts (6.1%-73.3% for ID and 3.6%-36.8% for autism spectrum disorders). The 42.3% and 14.1% prevalence of clubfoot and Poland anomaly, respectively, are also consistent with prior studies (28.0%-53.0% and 2.0%-38.2%). Although major limb anomalies occurred in 31.5% of our cohort, direct comparisons with previous reports are challenging because of differing definitions of limb anomalies.

A cluster analysis by Bell et al^16^ of 449 MBS cases identified by systematic literature review, with minimum diagnostic criteria of both CN6 and CN7 affected, identified 2 subgroups: one with “less severe” features (feeding or swallowing difficulties, micrognathia, and limb abnormalities including Poland syndrome) and another with more severe features (failure to thrive, developmental delay, additional CN palsies, and palatal and brain abnormalities upon imaging or at autopsy). Although our cluster analysis did not reveal distinct subclusters within our data set, comparing co-occurrence of various features revealed notable associations, with some overlap with the findings of Bell et al.^16^ Individuals with cleft palate, vocal cord paralysis/tracheostomy, and gastrointestinal anomalies/gastrostomy were more likely to exhibit neurological phenotypes of ID, autism, or seizures. In contrast, limb defects/Poland anomaly, micrognathia, and scoliosis were not more frequently observed in those with these neurological symptoms. The lack of strong subdivisions within MBS highlights the complexity of providing accurate prognostic information and genetic counseling to individuals and their families. Further research has the potential to validate and expand upon our phenotypic findings.

Despite comprehensive analyses of ES and GS data from 67 individuals with MBS, we did not identify any compelling or recurrent variants that indicate a germline genetic cause. Only 2 genes, *TTN* (HGNC:12403) and *COL24A1* (HGNC:20821), were mutated in more than 1 proband, and both were deprioritized because of their large genomic size, lack of missense constraint, and absence of embryonic CN6/CN7 expression. Additionally, the phenotypes of our probands were inconsistent with those reported with *TTN* variants. Beyond these 2 genes, we highlight variants in 12 candidates, but their role in MBS remains speculative. Although some candidate genes identified in our study could be considered compelling for biological reasons, each was found in only a single family. Confirming their involvement would require the identification of variants in additional MBS probands and functional validation. Some individuals in our cohort exhibited phenotypes overlapping known neurodevelopmental disorders associated with these candidate genes. However, no previously reported cases with variants in these genes are known to have MBS. The absence of convincing causative pathogenic variants in our cohort aligns with our finding of no statistically significant difference in DNV rates between MBS and non-MBS cohorts and with recent studies reporting negative ES findings in 18 and 30 probands with MBS, respectively.^29^^,^^30^ Furthermore, both these studies in addition to ours did not identify variants in *PLXND1* or *REV3L*, previously reported to cause MBS.^25^ This outcome suggests that, if germline variants in these genes do, indeed, cause MBS, they do so extremely rarely. Notably, in contrast to our strict participant selection, the studies by Moresco et al^30^ and Tomas-Roca et al^25^ reported variants in candidate genes in individuals who did not meet MBS diagnostic criteria, such as individuals without CN6 palsy (isolated congenital facial weakness) or with oculomotor nerve/CN3 involvement (CFEOM diagnostic spectrum). This underscores the importance of applying strict inclusion criteria to facilitate comparisons between cohorts and prevent confusion that could affect genetic counseling and testing for affected individuals and their families. Finally, although it remains plausible that MBS could be caused by noncoding pathogenic variation, the absence of vertical or horizontal transmission in our cohort suggests against any highly penetrant germline variation as a primary etiology.

Given the lack of a definitive highly penetrant germline genetic etiology, alternative mechanisms for MBS must be considered. One possibility is a weakly heritable oligogenic model in which variants in multiple genetic loci, each with relatively modest individual contributions, may have a cumulative impact on protein-protein interactions, or affect pleiotropic signaling pathways controlling rhombomere development resulting in the manifestation of MBS.^47^, ^48^, ^49^, ^50^ Alternatively, polygenic risk factors resulting from poorly understood multiple gene or gene-environment interactions (ie, hypoxia and vascular disruptions)^51^, ^52^, ^53^ or combined effects of rare and common variants may contribute to MBS pathogenesis as has been seen with some rare birth defects or neurodevelopmental disorders.^54^ The increasing recognition of postzygotic pathogenic variants causing a variety of developmental disorders raises the possibility that MBS could arise from a pathogenic somatic variant occurring de novo during embryonic development in a critical cell population in the brainstem.^55^ However, identifying such variants amid hypo/dysplastic tissue presents challenges, and future studies will require innovative sequencing approaches to address this question.

In the absence of a clear germline etiology for MBS, it is important to revisit environmental causes, which have long been proposed to contribute to MBS pathogenesis. Infectious etiologies have been posited, although no viral inclusions have been identified on brain autopsies of individuals with MBS.^56^ Prenatal exposure to drugs known to cause vasoconstriction and/or increased uterine tone such as misoprostol,^20^ ergotamine,^22^ thalidomide,^24^ and cocaine,^21^ alongside other teratogens with similar mechanisms, has been associated with MBS. In our cohort, 2 probands had exposure to misoprostol and notably, in these individuals, we did not identify genetic candidates of interest. Uterine shape or position, prenatal hypoxia or trauma, twinning, premature birth, or low birth weight have also been suggested. Mechanical trauma from uterine contraction may lead to maldevelopment because the developing CN6 and CN7 nuclei are located at a level in the embryo that would be compromised with pressure in the cephalocaudal direction.^57^ Moreover, given the location of the hindbrain within a vascular watershed, premature regression of the trigeminal artery or disruption of the vertebral supply could lead to brainstem ischemia and subsequently MBS. More extensive ischemia or hemorrhage may explain variable additional CN palsies. MBS with pectoralis hypoplasia has been suggested to result from a more proximal vascular event at the level of the subclavian artery.^17^ Focused arterial imaging studies could help explore the role of vascular malformations/disruptions outside the brainstem in the etiology of talipes equinovarus and transverse limb reduction defects in subsets of individuals with MBS. Vascular disruption or teratogens have been similarly postulated in the pathogenesis of other sporadic congenital oromandibular hypogenesis and limb malformation syndromes that exhibit phenotypic overlap with MBS.^40^^,^^41^ Consistent with these theories, we previously reported a small region of volumetric reduction in the paramedian pontine reticular formation and the MLF, important structures for conjugate horizontal gaze, that differentiates individuals with MBS from individuals with isolated CFP and from controls.^31^ Moreover, the size of volume loss correlated with phenotype severity and the presence of additional neuro/neurocognitive and other system involvement. Whether these changes reflect primary maldevelopment of these anatomic structures or are secondary to a destructive process remains undetermined.

In conclusion, individuals with MBS exhibit high phenotypic variability, and a unifying germline genetic etiology now seems unlikely. Strict diagnostic criteria, supported when necessary by CN imaging and electrophysiology studies, will differentiate MBS from similar conditions. Although germline variants in *PLXND1* (HGNC:9107) and *REV3L* (HGNC:9968) may represent extremely rare causes of MBS and related disorders, the absence of affected family members in our cohort in combination with our genetic studies suggest that MBS is most likely non-Mendelian in origin. Given the low diagnostic yield of germline ES/GS, future research should prioritize investigation into somatic genetic changes, environmental factors, or multifactorial models of disease. Our findings provide important insights for clinical geneticists, aid in genetic counseling and individualized care, and should help shape future research directions.

## Data Availability

The data/analyses presented in the current publication have been deposited in and are available from the dbGaP database under dbGaP accession numbers phs001383.v1.p1 and phs001247.v1.p1.

## Conflict of Interest

Silvio Alessandro Di Gioia is currently an employee and stockholder of Regeneron Pharmaceutical. Ke Hao received compensation as a part time employee of GeneDx LLC and is a stockholder with the company. Flavia M. Facio is currently an employee and stockholder of Invitae Corp. Sherin Shaaban is currently an employee of ARUP Laboratories. All other authors declare no conflicts of interest.
