# Supplementary material for: Systematic phenotype and genotype characterization of Moebius syndrome
Source: Genet Med Open. 2025 May 19;3:103437. doi: 10.1016/j.gimo.2025.103437 (PMC12256340; doi:10.1016/j.gimo.2025.103437)
Supplement: Supplementary Material [file mmc2.docx]

**SUPPLEMENTARY INFORMATION**

**Index:**

[**1.** **Supplementary Phenotyping Methods** 2](#_Toc185638279)

[i. Participant enrollment and phenotyping 2](#_Toc185638280)

[ii. Excel table of MBS cohort phenotype features 2](#_Toc185638281)

[**2.** **Supplementary Genotyping Methods** 2](#_Toc185638282)

[i. Quality control of sequence reads 2](#_Toc185638283)

[ii. Assessment of DNA contamination 3](#_Toc185638284)

[iii. Sequence alignment, variant calling, and prioritization of SNVs/indels 3](#_Toc185638285)

[iv. Concordance of genotypes called using ES and GS and GATK and Octopus callers 4](#_Toc185638286)

[v. Genotype concordance with expected family relationships and sex 4](#_Toc185638287)

[vi. Estimation of genetic ancestry of the sequenced probands 4](#_Toc185638288)

[vii. Calculation and application of variant minor allele frequency cutoffs 5](#_Toc185638289)

[viii. *De novo* variant rate analysis 5](#_Toc185638290)

[ix. Structural variant (SV) calling using GS data 6](#_Toc185638291)

[x. SV filtering for candidate pathogenic variants 7](#_Toc185638292)

[**3.** **Supplementary Results** 7](#_Toc185638293)

[i. Additional prenatal history details 7](#_Toc185638294)

[ii. Concordance of genotypes called using ES and GS and GATK and Octopus callers 7](#_Toc185638295)

[iii. Genotype concordance with expected family relationships and sex 8](#_Toc185638296)

[iv. Estimation of genetic ancestry of the sequenced probands 8](#_Toc185638297)

[v. Additional details on prioritized candidate genes submitted to Matchmaker exchange 8](#_Toc185638298)

[vi. Other genetic observations 9](#_Toc185638299)

[vii. DNV analysis 9](#_Toc185638300)

[viii. *PLXND1/REV3L* analysis 10](#_Toc185638301)

[**4.** **Supplementary References** 11](#_Toc185638302)

# **Supplementary Phenotyping Methods**

## **Participant enrollment and phenotyping**

Participants enrolled to the protocol had standardized evaluations at the clinical research center including: a physical/dysmorphology exam and genetic counseling session; ophthalmology examination with gross eye movement video recordings as well as videonystagmography to evaluate and record oculomotor function and static and dynamic eye movements; neurology examination with neurophysiology testing including electromyogram and nerve conduction studies and head MRI with diffusion tensor imaging (DTI); craniofacial evaluation including 3D facial imaging and cone-beam CT; audiology examination for diagnostic hearing assessments; physiatry assessment with additional assessment by physical, occupational, and speech/language therapists; and neurodevelopmental assessments by a team of neuropsychologists and psychiatrists using age-appropriate instruments, including diagnostic autism screening.

MRI findings are documented in Supplementary Table 4. Because the facial muscles are best resolved with high quality axial T1-weighted images acquired from above and below the chin, and because the normal size ranges of the facial muscles are not rigorously validated in pediatric populations, we did not assess these muscles on MRI.

## **Excel table of MBS cohort phenotype features**

Phenotype data collected in REDCap were extracted and summarized in Supplementary Table 1. This table includes sample ID, demographics (age at evaluation, sex assigned at birth, and self-reported race/ethnicity), whether sequencing was performed, characterization of facial weakness and eye movement anomalies, and 29 additional phenotypic features and/or system involvement. Statistical analysis of phenotypic features was performed using IBM® SPSS Statistics version 21 (Chicago, IL, USA) or GraphPad Prism version 7.0c (Carey, North Carolina, USA).

# **Supplementary Genotyping Methods**

We generated exome and/or genome sequencing (ES, GS) data for 184 individuals (67 probands and 117 unaffected family members), with 6 individuals having both ES and GS data sets. Library preparations for ES were primarily performed at the National Human Genome Research Institute with sequencing completed at the NIH Intramural Sequencing Center. DNA libraries were prepared for ES using Nimblegen SeqCap EZ Exome v2 (Roche) or SureSelect Human All Exon v4 kit (Agilent) and sequenced either on Illumina Hiseq 2000 or Hiseq 2500. Genome sequencing (GS) was completed for 27 trios through the NIH Gabriella Miller Kids First (GMKF) Pediatric Research Program at the Baylor College of Medicine Human Genome Sequencing Center (Houston, TX).

## **Quality control of sequence reads**

On average, 729 and 774 million reads per sample were generated by ES and GS, respectively. All samples had ≥98% of exonic regions (ccdsGene, genome.ucsc.edu), with a mean sequencing depth of 113x (>=44x) (Supplementary table 6). For the 6 probands with both ES and GS, there was 99.47% concordance among 9,540 chromosome 22 SNVs, and ES were used for downstream analyses. We assessed sequence quality of the paired end reads with FastQC (v0.10.0, <http://www.bioinformatics.babraham.ac.uk/projects/fastqc/>) for each of 40 ES and 54 GS. We used MultiQC (PMID: 27312411) (v1.8) to summarize the FastQC results. We evaluated the base quality scores (“per_base_sequence_quality_scores”) and sequence quality scores (“per_sequence_quality_scores”), and detected all samples passed MultiQC thresholds with “pass”, indicating high quality of the sequencing data.

## **Assessment of DNA contamination**

We assessed potential contamination of DNAs used for ES and GS using verifybamID (v1.1.1)^1^. We extracted uniquely aligned reads in the exome captured regions for ES and GS data after excluding PCR duplicates. Next, we ran verifybamID with default options except “--ignoreRG –precise” with 1000G phase 3.v5 allele frequency data (https://www.internationalgenome.org/data/) as the reference SNP allele frequency vcf file. With the population allele frequency information of 173,767 SNPs in the exome captured regions, we did not detect any samples with contamination (FREEMIX < 0.05).

## **iii. Sequence alignment, variant calling, and prioritization of SNVs/indels**

Paired-end sequence reads were mapped to the human reference genome GRCh37 using 'bwa mem' (v0.7.17)^2^. Germline SNVs and indels were called following recommendations of the GATK best practice variant calling guidelines^3^ in the tiled regions of the exome kits. Briefly, we sorted the aligned reads with ‘gatk’ (4.0.5) 'SortSam', followed by flagging the duplicate reads with 'MarDuplicates'. We recalibrated base quality scores of the aligned reads using a model developed from 1000G known indels ([https://www.internationalgenome.org](https://www.internationalgenome.org/)) and dbSNP 151 SNPs (<https://www.ncbi.nlm.nih.gov/snp/>).

Subsequently, we determined the genotype likelihood of each sample at a given site and assigned most likely genotypes by the ‘gatk’ 'HaplotypeCaller' function in tiled regions of exome sequencing kits (SeqCap EZ Exome v2.0 or xGen Exome Research Panel v1, Integrated DNA Technologies, IDT). Next, we jointly called genotypes for ES and GS using the 'GenotypeGVCFs' function.

We constructed variant recalibration models separately for SNVs and indels using 'VariantRecalibrator'. For SNVs, we considered the following set of known variants for the recalibration: SNPs included in 1000G phase 1, HapMap, Illumina Omni2.5 chip, and dbSNP 151 database (including indels) (<https://www.ncbi.nlm.nih.gov/snp/>) with options '--trust-all-polymorphic -tranche 100.0 -tranche 99.95 -tranche 99.9 -tranche 99.8 -tranche 99.6 -tranche 99.5 -tranche 99.4 -tranche 99.3 -tranche 99.0 -tranche 98.0 -tranche 97.0 -tranche 90.0 -an QD -an MQRankSum -an ReadPosRankSum -an FS -an MQ -an SOR -an DP --max-gaussians 6'. Similarly, for indels, we used variants included in Affymetrix Axiom Exome chip, dbSNP 151 database and 1000G phase 1 for the variant recalibration with additional parameters: ‘-tranche 100.0 -tranche 99.95 -tranche 99.9 -tranche 99.5 -tranche 99.0 -tranche 97.0 -tranche 96.0 -tranche 95.0 -tranche 94.0 -tranche 93.5 -tranche 93.0 -tranche 92.0 -tranche 91.0 -tranche 90.0 --max-gaussians 4 -an FS -an ReadPosRankSum -an MQRankSum -an QD -an SOR -an DP'. Finally, we applied the models to the newly generated variant genotypes with the ‘ApplyVQSR’ function and assigned quality scores.

Variant calls for the 52 trios and 3 quads were filtered with Qiagen QCI Interpret based on confidence (call quality ≥69, genotype quality ≥20, allele fraction ≥20, read depth >10, and outside top 5% most exonically variable 100 base windows in healthy public genomes); population frequency (MAF<0.001% for X-linked recessive and de novo filtering; MAF <0.5% for autosomal recessive filtering); predicted deleteriousness (retained frameshifts, in-frame indels, start/stop codon changes, missense, and splice site loss variants up to 2 bases into the intron or as predicted by MaxEntScan, or listed in HGMD); and mode of inheritance (de novo or recessive filtering) (Qiagen Redwood City, [https://digitalinsights.qiagen.com](https://digitalinsights.qiagen.com/)). Genes with frequent variation in the population (olfactory receptor, HLA, and mucin genes), variants in repetitive genomic regions, variants resulting in amino acid addition or deletion in a tract of ≥8 of the same amino acid, and indels within 100 bp of other indels were excluded. Sequence alignments were inspected manually using ‘samtools tview,’ and variants on poorly aligned reads with >1 neighboring mismatching alleles were also excluded. Variant call data for 12 additional probands without one or both parents were reviewed for variants in candidate genes identified in trios. All probands were reviewed for variants in candidate genes identified from the literature.

## **iv. Concordance of genotypes called using ES and GS and GATK and Octopus callers**

We generated ES data for 184 individuals, 67 probands and 117 family members, with 6 probands having both ES and GS data sets. We compared the genotypes called between the two data sources. Similarly, we used Octopus (v0.6.3-beta) to call genotypes for 184 samples using bwa alignment bam files. We identified SNVs/indels in each sample individually in the tiled regions of the exome kits.

## **v. Genotype concordance with expected family relationships and sex**

We pruned biallelic SNVs called in the exome captured regions of 184 individuals using plink (v1.90b2m)^4^ with options “--indep-pairwise 50 5 0.5” (in a sliding window of 50 SNPs with a shift of 5 SNPs at each step, removing duplicate SNPs with r^2 > 0.5). We assessed the consistency of family relationships using king^5^ (v1.4). Next, we used plink with an option “--check-sex” to verify sex of the samples.

## **vi. Estimation of genetic ancestry of the sequenced probands**

Using ES/GS, ancestries were nominated for the 67 sequenced probands by using GRAF-pop^6^. GRAF-pop calculates genetic distances of each individual from three reference populations (European, Asian, and African) compiled from the database of Genotype and Phenotypes (dbGaP) using a set of “fingerprint” SNPs. Ancestry is inferred from the genetic distance scores from each reference population. A mean of 3,296 SNVs per individual were used for the prediction. (Supplementary Figure 4).

## **vii. Calculation and application of variant minor allele frequency cutoffs**

Previous studies have estimated that MBS has a prevalence of 0.3:100,000-1:50,000 (0.000003-0.00002)^7,8^. The lower end of this range is a more recent estimate based on the 2007 revised MBS diagnostic criteria, and is likely closer to the true population frequency of MBS after removing individuals with common misdiagnoses (e.g., those with *TUBB3*-related syndromes). To calculate minor allele frequency cutoffs for variant filtering, we selected a MBS prevalence estimate of 0.000005 which is intermediate between these two values but closer to the lower end of the range.

Under a de novo variant model, the allele frequency cutoff for de novo variants is equal to the population frequency of the condition itself (0.000005). We rounded this value upward to obtain a minor allele frequency cutoff of 0.00001 (i.e. 0.001%) for de novo variant filtering. To obtain allele frequency cutoffs under an autosomal recessive variant model, we applied the Hardy Weinberg equation (p+q=1; p2 + 2pq +q2=1).^9^ Assuming the population frequency of MBS defined above, q2 (the frequency of people with the recessive phenotype) is 0.000005, and the frequency of the variant allele q is 0.002236. The carrier frequency for the variant allele in the population (2pq) = 2*.99774*.002236 = .00447. We rounded this value up to 0.005 (i.e. 0.5%), which is the value we used as our cutoff for autosomal recessive variants.

To obtain allele frequency cutoffs under an X-linked recessive variant model, we applied the Hardy Weinberg equation for X-linked variants9 (Table 1). Under this model, assuming that MBS affects males and females at equal rates, the prevalence of MBS in the population equals φpA + (1−φ) pA2, where pA is the frequency of the X-linked variant allele and φ̂ is the fraction of males in the population (~0.5). Therefore, 0.5*pA + 0.5*pA2=0.000005, resulting in an estimate of pA=0.00001 (i.e. 0.001%).

For X-linked and autosomal de novo filtering and for X-linked recessive filtering, variants were excluded if ≥0.001% of subpopulation with highest allele frequency in gnomAD v2.1.1 or ExAC^10^, or ≥0.001% of appropriate population in NHLBI ESP exomes, or ≥0.001% in the 1000 Genomes Project, unless as established as a pathogenic common variant. For autosomal recessive filtering, variants were excluded if 0.5% of subpopulation with highest allele frequency in gnomAD v2.1.1 or ExAC^10^, or ≥0.5% of appropriate population in NHLBI ESP exomes, or ≥0.5% in the 1000 Genomes Project, unless the variant was established as a pathogenic common variant.

## **viii. *De novo* variant rate analysis**

To determine whether individuals with MBS have increased rare coding/splice site *de novo* SNVs (DNVs), DNVs from our 55 MBS trios/quads (28 ES, 27 GS) were analyzed in parallel to published DNVs from GS of 1,449 trios from the TOPMed Program^11^. Note that for three of the 55 families used for DNV analyses, we had sequenced an additional unaffected sibling of the proband (included in quad sequencing counts in the main manuscript) but excluded the sibling’s sequence data for these analyses, so only the trio was considered. We analyzed both datasets with Qiagen QCI Interpret (Qiagen Redwood City, [https://digitalinsights.qiagen.com](https://digitalinsights.qiagen.com/)) using the DNV frequency filters defined above and retained SNVs predicted to result in autosomal missense, start/stop codon changes, or splice site loss up to 2 bases into the intron or as predicted by MaxEntScan. Sequence alignments of variants in our MBS dataset were inspected manually to obtain a more conservative estimate of variant numbers. This filtered MBS dataset was compared to additional published ES-derived DNVs from 1,789 trios comprising parents and unaffected siblings of probands from the Simons Simplex Consortium (SSC; variant list obtained from supplementary dataset S10 in Jin et al^12^; original control dataset described in Krumm et al^13^. This dataset was filtered using the same frequency and annotation filters described above. The numbers of DNVs per individual were counted. DNV rates in MBS versus each control population were compared by the Mann-Whitney two-sided U test.

To control for different sequencing methods or processing pipelines, we compared GS of 29 MBS trios to 40 non-MBS control trios sequenced in parallel through GMKF and called jointly (dbGaP accession number phs001247.v1.p1; sequencing and processing of this cohort summarized in Jurgens et al^14^, Family 1). The 29 MBS trios included 27 trios who had only GS and 2 additional trios who had both ES and GS, but for whom GS was used in this analysis. The 40 non-MBS trios included 10 trios comprised of unaffected parents and an unaffected sibling of individuals with CCDDs; 22 non-MBS trios with other CCDDs solved by *de novo* variants in known monogenic CCDD genes; and 8 non-MBS CCDD trios solved by a heterozygous variant in a known monogenic CCDD gene with an autosomal dominant transmission pattern. Trios were analyzed for autosomal DNVs predicted to result in missense, start/stop codon changes, or splice site loss up to 2 bases into the intron with MAF<0.00001 in public datasets and MAF<0.01 in our call set, to exclude false positives among jointly called samples. Variants that passed call quality filters with genotype quality >20 and allele balance>20 were retained. The numbers of DNVs per individual were counted, and paternal ages at conception were obtained for each trio. DNV rates in the two populations were compared by the Mann-Whitney two-sided U test and by Poisson regression using the dataset (MBS or control) and paternal age at conception as covariates. Poisson test power calculations were performed using PASS 2022 Power Analysis and Sample Size Software (NCSS, LLC., Kaysville, Utah, USA, ncss.com/software/pass), assuming sample sizes of 29 and 40 individuals in the MBS and GMKF control cohorts, respectively, using a two-sided test of the difference between Poisson rates, assuming a significance level of 0.05, and using the observed mean DNV rates in the two cohorts.

## **ix. Structural variant (SV) calling using GS data**

An SV calling and genotyping pipeline was developed and applied to the GS data for SV analysis. This pipeline uses an ensemble learning strategy consisting of three major steps: 1) calling SVs with multiple tools; 2) merging the different SV callsets into consensus SV sites for each individual and then across individuals; 3) joint genotyping each SV site across individuals. SV calls from each individual were generated using 11 state-of-the-art SV callers: Manta^15^, LUMPY^16^, BreakDancer^17^, BreakSeq2^18^, cn.MOPS^19^, CNVnator^20^, DELLY^21^, GenomeSTRiP^22^, Hydra^23^, MELT^24^, and TIGRA^25^. The tool selection is based on population-scale SV studies^26^; bioRxiv 2020.05.02.074096. These methods use read depth, discordant read pairs, and split reads to identify SV location and breakpoints up to single base-pair resolution. Most of them detect multiple types of SVs while a few are specialized at detecting specific SV types. For each individual, initial SV calls were stored in multiple VCF files, one for each caller, and then merged into consensus SV sites using SURVIVOR^27^ using the following criteria: >50 bp in length; supported by at least 2 callers with a maximum discrepancy of 50 bp in breakpoints; and agreeing on type and strand. The consensus SVs in each individual were genotyped using SVTyper^28^, and those with missing or homozygous reference genotypes were excluded. SVs from multiple individuals were combined using SURVIVOR and jointly genotyped using SVTyper. For samples collected from families, SVs at the individual level were combined and genotyped at the family level before being processed at the cohort level. This “bottom-up” strategy reduces false positives while maintaining sensitivity.

## **x. SV filtering for candidate pathogenic variants**

Following SV calling and genotyping, the resulting SVs from each family were subjected to a series of filtering criteria to identify and prioritize potential candidate pathogenic variants. SVs stored in family-wise VCFs were first filtered by family-specific inheritance models (autosomal dominant, de novo, and recessive). The remaining SVs were annotated with allele frequency from public SV databases including 1000 Genomes Project^29^ and gnomAD-SV^26^ as well as in-house SV datasets based on GS data generated from healthy controls in the studies of Gabriella Miller Kids First Pediatric Research (Kids First) program, (dbGaP accession numbers: phs001247.v1.p1, phs001168.v2.p2, phs001138.v3.p2, and phs001178.v1.p1), processed with the same SV calling pipeline for this study. SVs were filtered if their variant allele frequency annotated from either public or internal datasets are greater than 0.1% for the dominant or de novo models or greater than 1% for the recessive model. The remaining SVs were further functionally annotated by AnnotSV^30^, followed by manual review and curation to generate a prioritized candidate list.

# **Supplementary Results**

# **i. Additional prenatal history details**

Nine cases were the product of a multiple pregnancy; six had a living unaffected fraternal twin; and 3 had a prenatal history positive for a vanishing fetus (Fig 3D, E).

## **ii. Concordance of genotypes called using ES and GS and GATK and Octopus callers**

The average genotype agreement across the samples that had both ES and GS was 99.47% (98.88-99.68%) out of 9,540 SNVs (9,200-9,543) for chromosome 22, indicating agreement of genotypes from two independent sets of sequencing data. For consistency, we used ES-derived genotypes for downstream analyses in instances where both data types were available, except for the comparison of DNV rate analyses of GMKF-sequenced GS datasets, as described (Supplementary Methods, section viii).

Comparison of genotypes of chromosome 22 indicates the SNVs/indels called by Octopus are highly concordant with those determined by GATK with an average rate of 99.92% (99.34-100%) out of an average of 994 SNPs per sample with at least one variant allele. These results suggest that our genotype calling is highly accurate. For all downstream analyses, we used genotypes prepared with the GATK best practice pipeline.

## **Genotype concordance with expected family relationships and sex**

We identified two nuclear families where the SNP genotypes called from the exomes do not support expected pedigree relationships. We excluded these families from downstream analyses (F3 and F37, six individuals). In addition, we identified 3^rd^ degree relatedness of parents of a proband (F36-001). We identified and corrected the identities of two samples that were switched (F93-002 and F93-003, parents of a proband F93-001) and reassigned the correct identities to corresponding DNAs. No additional problems were detected.

## **Estimation of genetic ancestry of the sequenced probands**

The population included 2 African, 3 Asian, 49 European, 12 Hispanic, and 1 individual with multiple ancestries. Inferred and self-reported ancestries were concordant in 61/67 individuals (91.0%) (Supplementary Table 7, Supplementary Figure 4).

## **Additional details on prioritized candidate genes submitted to Matchmaker exchange**

Three genes encode deubiquitinating enzymes (*USP15* [HGNC:12613]*, MINDY1* [HGNC:25648]*,* and *ZRANB1* [HGNC:18224]*)*^31-33^, and KPNA3 [HGNC:6396] facilitates the nuclear import of the deubiquitinase ATXN3 [HGNC:7106]^34^. Among these, a *de novo* missense variant in *ZRANB1* was previously reported in an individual with facial weakness, microcephaly, constipation, seizures, tongue fasciculations, and intracranial calcifications; however, no horizontal gaze restriction was reported, and the mode of inheritance differs from F75-001, who harbors compound heterozygous *ZRANB1* variants^35^. Morpholino knockdown of a zebrafish *ZRANB1* ortholog leads to decreased expression of neuronal marker huC and abnormal neuronal patterning^36^.

*MORC2* [HGNC:23573] was prioritized because *Morc2*^-/-^ mice have abnormal topology of the facial, hypoglossal, and vagus nerves, abnormal morphology of the olfactory nerve, absent mandibular nerves, and absent eye muscles (MGI Reference ID: J:237616). Pathogenic heterozygous *MORC2* missense variants are known to cause an axonal polyneuropathy with or without developmental delay^37,38^, and three affected individuals were also reported to have variable degrees of facial weakness^39,40^ (<https://zenodo.org/record/6388227#.YvPcBy1h3q1>), but none had accompanying horizontal gaze restriction consistent with MBS. Their dominant mode of inheritance also differs from F79-001, who has compound heterozygous missense *MORC2* variants.

*STMN3* [HGNC:15926] was prioritized because it encodes a microtubule-destabilizing protein^41,42^; pathogenic variants in *TUBB3* [HGNC:20772], which encodes a beta-tubulin monomer that is a component of microtubules, can cause paralytic strabismus and CFP in individuals misdiagnosed with MBS, and these variants increase microtubule stability^43^. *PBXIP1* [HGNC:21199] was prioritized because pathogenic variants in the homeodomain protein *HOXB1* [HGNC:5111] cause CFP^44^, and PBXIP1 inhibits DNA binding of HOXB1-PBX1 heterodimers^45,46^.

*PALM* [HGNC:8594] encodes for a neuronal protein involved in cytoskeletal organization and branching and filopodial formation in axons and dendrites^47^.

## **Other genetic observations**

A *PIEZO2* [HGNC:26270] DNV c.1535G>A, p.(Ser512Asn) (NM_001378183.1; NM_022068.4) was identified in F32-001 with bilateral ocular abduction deficits with normal adduction, severe bilateral CFP, tongue hypoplasia, left-sided pectoralis muscle hypoplasia, ipsilateral hand brachydactyly, flexed shortened thumb, bilateral clubfeet, and flexed 3rd toes. This individual had no ptosis, arthrogryposis, or scoliosis (Supplementary Table 1). One ClinVar submission classified this as a variant of uncertain significance for an undescribed phenotype (VCV000432484.2), but otherwise, it has not been described in the literature. Heterozygous gain-of-function variants in *PIEZO2* located primarily in the intracellular C-terminal domain of PIEZO2 cause various phenotypes. These include dominant distal arthrogryposis type 3 associated with cleft palate (Gordon syndrome); distal arthrogryposis type 5, associated with ptosis, ophthalmoplegia, Duane syndrome^48^, and restrictive lung disease; and Marden-Walker syndrome, associated with mask-like facies, blepharophimosis, micrognathia, cleft palate and arthrogryposis^49,50^. Despite some overlapping features (clubfeet, mild brachy/camptodactyly and flexed/shortened thumb in the hand ipsilateral to pectoralis hypoplasia, and mask-like facies), other features of distal arthrogryposis 5 differ from our case, including the contracture pattern, age-dependent progression, and ptosis/ophthalmoplegia.

A heterozygous c.103T>C, p.(Tyr35His) variant in *CHRNE* [HGNC:1966] (NM_000080.4) was identified in F37-001 and absent in the unaffected mother of the proband. The father was not sequenced. The phenotype of F37-001 is not consistent with *CHRNE-*related myasthenia, suggesting that this variant may not be causal.

A heterozygous c.278delG, p.(Ser93fs*3) variant in *MEPE* [HGNC:13361] (NM_001184697.1) was identified in F5-001 and was inherited from an unaffected parent. While this variant was reported as causal for HCFP and otosclerosis^51^, F5-001 does not have otosclerosis, and has limited abduction, bilateral brachysyndactyly, and absent left foot, suggesting this variant may not cause that phenotype, at least not under a fully penetrant autosomal dominant inheritance model.

## **DNV analysis**

We identified mean numbers of rare coding or splice site de novo variants (DNVs) per individual of 0.41, 0.55, and 0.67 in the 1,449 TOPMed trios, 1,789 SSC trios, and our 55 MBS trios/quad-derived trios, respectively. DNV rates differed significantly between MBS versus TOPMed but not MBS versus SSC cohorts, (p=0.0005, 0.071, respectively; Mann-Whitney two-sided U-test; Supplemental Figure 5A-B), but we suspected that the former difference may be attributable to factors unrelated to MBS status such as sequencing or variant calling methodologies. To test this, we compared the TOPMed versus SSC cohorts and found that the two cohorts had significantly different rates (p-value = 3.657e-08; Mann-Whitney two-sided U-test; Supplemental Figure 5C), suggesting that these technical variables unrelated to MBS status may play a role in differential DNV rates between cohorts.

To test for the effects of different sequencing platforms in our MBS cohort, we compared DNV rates in probands sequenced by ES (n=28) versus GS (n=27) and found no significant difference (mean numbers of 0.64 variants per individual and 0.70 variants per individual for ES and GS, respectively; p=0.664, Mann-Whitney two-sided U-test; Supplemental Figure 5D).

To control for differences in sequencing and processing methods, we compared GS of 29 MBS trios to 40 non-MBS GMKF control trios sequenced in parallel and called jointly. The mean number of DNVs per individual did not differ between these cohorts (mean of 0.88 and 0.76 variants per individual, respectively; p=0.9843, Mann-Whitney two-sided U-test; Supplemental Figure 5E).

To control for differences in paternal age at conception, we performed Poisson regression to model the number of DNVs per individual based on the dataset (MBS versus non-MBS GMKF cohort) and on paternal age at conception. Mean paternal age at conception for the 29 genomed MBS trios was 29.76 years (SD 5.15 years); four (13.8%) had paternal ages >35 years. Neither dataset nor paternal age had a statistically significant impact on DNV rate; the MBS cohort had fewer DNVs per person than the non-MBS GMKF cohort, but this difference is not statistically significant (p = 0.856), and there is a 3% increase in DNVs per individual for each additional year in paternal age, but this does not contribute to a statistically meaningful difference (p =0.109). Note that the small sample sizes of the two cohorts (n=29 and 40) has only 8.49% power to detect differences in DNV rates per individual using a two-sided test of the difference between Poisson event rates, assuming a significance level of 0.05 and that mean DNV rates are as observed in the two cohorts.

## ***PLXND1* [HGNC:9107] and *REV3L* [HGNC:9968]** **analysis**

Tomas-Roca *et al* presented three probands with rare *PLXND1* DNVs (two missense, one synonymous)^52^ (in their Supplementary Table 3). There are distinct missense substitutions at the same residues as the two reported missense variants in gnomAD^10^, no disease mechanism was proposed for the synonymous variant, and functional studies were not performed to demonstrate these variants’ pathogenicity. Moreover, the phenotypes reported in these individuals were variable and sometimes more consistent with non-MBS phenotypes such as CFEOM (Supplementary Table 5, P9). Although *Plxnd1*-/- mice have decreased numbers and aberrant migration of facial motoneurons, the *de novo* variants reported in humans were not shown to be loss of function^52^, and facial and eye movement were not analyzed.

In addition, Tomas-Roca et al. presented three probands with *de novo* *REV3L* variants, including a splice site, missense, and stopgain variant which are absent from gnomAD^10^. The phenotypes of the reported individuals were variable and in one case more reminiscent of CFP than MBS (Supplementary Table 5, P12). Functional studies were performed for the splice site but not the missense or stopgain variant. *Rev3l+/-* mice at P0 had fewer motoneurons than wild-type mice^52^, but facial and eye movements were not analyzed. One additional report has described a heterozygous *PLXND1* missense variant in a single individual with MBS-Poland syndrome^53^, but the variant is inherited from the unaffected mother of the proband and is present in 21 individuals in gnomAD^10^, so pathogenicity is uncertain.

**viii. Maternal thrombophilia gene screen**

We looked for pathogenic/likely pathogenic variants in genes associated with increased risk for thrombophilia in available sequences among the mothers of 52 MBS probands. Eight mothers had 9 rare coding or splice site variants in maternal thrombophilia genes consistent with the known modes of inheritance for the associated conditions (Supplementary Tables 12,13). These included 7 heterozygous variants in 7 mothers (*TBXA2R* [HGNC:11608]*, RUNX1 [*HGNC:10471], and *VWF* [HGNC:12726]) and 2 compound heterozygous variants in 1 mother (*ADAMTS13* [HGNC:1366]). Of these mothers, only one (F160-003 with compound heterozygous *ADAMTS13* variants) had self-reported complications during pregnancy that were potentially consistent with the associated condition. These included premature labor and rupture of membranes in F160-003.

# **Supplementary References**

1. Jun, G. *et al.* Detecting and estimating contamination of human DNA samples in sequencing and array-based genotype data. *Am J Hum Genet* **91**, 839-48 (2012).

2. Li, H. & Durbin, R. Fast and accurate short read alignment with Burrows-Wheeler transform. *Bioinformatics* **25**, 1754-60 (2009).

3. Poplin, R. *et al.* Scaling accurate genetic variant discovery to tens of thousands of samples. *bioRxiv*, 201178 (2018).

4. Purcell, S. *et al.* PLINK: a tool set for whole-genome association and population-based linkage analyses. *Am J Hum Genet* **81**, 559-75 (2007).

5. Manichaikul, A. *et al.* Robust relationship inference in genome-wide association studies. *Bioinformatics* **26**, 2867-73 (2010).

6. Jin, Y., Schaffer, A.A., Feolo, M., Holmes, J.B. & Kattman, B.L. GRAF-pop: A Fast Distance-Based Method To Infer Subject Ancestry from Multiple Genotype Datasets Without Principal Components Analysis. *G3 (Bethesda)* **9**, 2447-2461 (2019).

7. Verzijl, H.T., van der Zwaag, B., Cruysberg, J.R. & Padberg, G.W. Mobius syndrome redefined: a syndrome of rhombencephalic maldevelopment. *Neurology* **61**, 327-33 (2003).

8. Carta, A. *et al.* The epidemiology of Moebius syndrome in Italy. *Orphanet J Rare Dis* **16**, 162 (2021).

9. Graffelman, J. & Weir, B.S. Testing for Hardy-Weinberg equilibrium at biallelic genetic markers on the X chromosome. *Heredity (Edinb)* **116**, 558-68 (2016).

10. Karczewski, K.J. *et al.* The mutational constraint spectrum quantified from variation in 141,456 humans. *Nature* **581**, 434-443 (2020).

11. Kessler, M.D. *et al.* De novo mutations across 1,465 diverse genomes reveal mutational insights and reductions in the Amish founder population. *Proc Natl Acad Sci U S A* **117**, 2560-2569 (2020).

12. Jin, S.C. *et al.* Contribution of rare inherited and de novo variants in 2,871 congenital heart disease probands. *Nat Genet* **49**, 1593-1601 (2017).

13. Krumm, N. *et al.* Excess of rare, inherited truncating mutations in autism. *Nat Genet* **47**, 582-8 (2015).

14. Jurgens, J.A. *et al.* Novel variants in TUBA1A cause congenital fibrosis of the extraocular muscles with or without malformations of cortical brain development. *Eur J Hum Genet* **29**, 816-826 (2021).

15. Chen, X. *et al.* Manta: rapid detection of structural variants and indels for germline and cancer sequencing applications. *Bioinformatics* **32**, 1220-2 (2016).

16. Layer, R.M., Chiang, C., Quinlan, A.R. & Hall, I.M. LUMPY: a probabilistic framework for structural variant discovery. *Genome Biol* **15**, R84 (2014).

17. Fan, X., Abbott, T.E., Larson, D. & Chen, K. BreakDancer: Identification of Genomic Structural Variation from Paired-End Read Mapping. *Curr Protoc Bioinformatics* **45**, 15 6 1-11 (2014).

18. Lam, H.Y. *et al.* Nucleotide-resolution analysis of structural variants using BreakSeq and a breakpoint library. *Nat Biotechnol* **28**, 47-55 (2010).

19. Klambauer, G. *et al.* cn.MOPS: mixture of Poissons for discovering copy number variations in next-generation sequencing data with a low false discovery rate. *Nucleic Acids Res* **40**, e69 (2012).

20. Abyzov, A., Urban, A.E., Snyder, M. & Gerstein, M. CNVnator: an approach to discover, genotype, and characterize typical and atypical CNVs from family and population genome sequencing. *Genome Res* **21**, 974-84 (2011).

21. Rausch, T. *et al.* DELLY: structural variant discovery by integrated paired-end and split-read analysis. *Bioinformatics* **28**, i333-i339 (2012).

22. Handsaker, R.E., Korn, J.M., Nemesh, J. & McCarroll, S.A. Discovery and genotyping of genome structural polymorphism by sequencing on a population scale. *Nat Genet* **43**, 269-76 (2011).

23. Lindberg, M.R., Hall, I.M. & Quinlan, A.R. Population-based structural variation discovery with Hydra-Multi. *Bioinformatics* **31**, 1286-9 (2015).

24. Gardner, E.J. *et al.* The Mobile Element Locator Tool (MELT): population-scale mobile element discovery and biology. *Genome Res* **27**, 1916-1929 (2017).

25. Chen, K. *et al.* TIGRA: a targeted iterative graph routing assembler for breakpoint assembly. *Genome Res* **24**, 310-7 (2014).

26. Collins, R.L. *et al.* A structural variation reference for medical and population genetics. *Nature* **581**, 444-451 (2020).

27. Jeffares, D.C. *et al.* Transient structural variations have strong effects on quantitative traits and reproductive isolation in fission yeast. *Nat Commun* **8**, 14061 (2017).

28. Chiang, C. *et al.* SpeedSeq: ultra-fast personal genome analysis and interpretation. *Nat Methods* **12**, 966-8 (2015).

29. Sudmant, P.H. *et al.* An integrated map of structural variation in 2,504 human genomes. *Nature* **526**, 75-81 (2015).

30. Geoffroy, V. *et al.* AnnotSV: an integrated tool for structural variations annotation. *Bioinformatics* **34**, 3572-3574 (2018).

31. Baker, R.T., Wang, X.W., Woollatt, E., White, J.A. & Sutherland, G.R. Identification, functional characterization, and chromosomal localization of USP15, a novel human ubiquitin-specific protease related to the UNP oncoprotein, and a systematic nomenclature for human ubiquitin-specific proteases. *Genomics* **59**, 264-74 (1999).

32. Kristariyanto, Y.A., Abdul Rehman, S.A., Weidlich, S., Knebel, A. & Kulathu, Y. A single MIU motif of MINDY-1 recognizes K48-linked polyubiquitin chains. *EMBO Rep* **18**, 392-402 (2017).

33. Zhang, P. *et al.* ZRANB1 Is an EZH2 Deubiquitinase and a Potential Therapeutic Target in Breast Cancer. *Cell Rep* **23**, 823-837 (2018).

34. Sowa, A.S. *et al.* Karyopherin alpha-3 is a key protein in the pathogenesis of spinocerebellar ataxia type 3 controlling the nuclear localization of ataxin-3. *Proc Natl Acad Sci U S A* **115**, E2624-E2633 (2018).

35. Deciphering Developmental Disorders, S. Large-scale discovery of novel genetic causes of developmental disorders. *Nature* **519**, 223-8 (2015).

36. Tse, W.K. *et al.* Genome-wide loss-of-function analysis of deubiquitylating enzymes for zebrafish development. *BMC Genomics* **10**, 637 (2009).

37. Guillen Sacoto, M.J. *et al.* De Novo Variants in the ATPase Module of MORC2 Cause a Neurodevelopmental Disorder with Growth Retardation and Variable Craniofacial Dysmorphism. *Am J Hum Genet* **107**, 352-363 (2020).

38. Jacquier, A., Roubille, S., Lomonte, P. & Schaeffer, L. Microrchidia CW-Type Zinc Finger 2, a Chromatin Modifier in a Spectrum of Peripheral Neuropathies. *Front Cell Neurosci* **16**, 896854 (2022).

39. Duan, X. *et al.* Characterization of genotype-phenotype correlation with MORC2 mutated Axonal Charcot-Marie-Tooth disease in a cohort of Chinese patients. *Orphanet J Rare Dis* **16**, 244 (2021).

40. Sevilla, T. *et al.* Mutations in the MORC2 gene cause axonal Charcot-Marie-Tooth disease. *Brain* **139**, 62-72 (2016).

41. Charbaut, E. *et al.* Stathmin family proteins display specific molecular and tubulin binding properties. *J Biol Chem* **276**, 16146-54 (2001).

42. Gavet, O. *et al.* The stathmin phosphoprotein family: intracellular localization and effects on the microtubule network. *J Cell Sci* **111 ( Pt 22)**, 3333-46 (1998).

43. Tischfield, M.A. *et al.* Human TUBB3 mutations perturb microtubule dynamics, kinesin interactions, and axon guidance. *Cell* **140**, 74-87 (2010).

44. Webb, B.D. *et al.* HOXB1 founder mutation in humans recapitulates the phenotype of Hoxb1-/- mice. *Am J Hum Genet* **91**, 171-9 (2012).

45. Piper, D.E., Batchelor, A.H., Chang, C.P., Cleary, M.L. & Wolberger, C. Structure of a HoxB1-Pbx1 heterodimer bound to DNA: role of the hexapeptide and a fourth homeodomain helix in complex formation. *Cell* **96**, 587-97 (1999).

46. Ferretti, E. *et al.* Hoxb1 enhancer and control of rhombomere 4 expression: complex interplay between PREP1-PBX1-HOXB1 binding sites. *Mol Cell Biol* **25**, 8541-52 (2005).

47. Gauthier-Campbell, C., Bredt, D.S., Murphy, T.H. & El-Husseini Ael, D. Regulation of dendritic branching and filopodia formation in hippocampal neurons by specific acylated protein motifs. *Mol Biol Cell* **15**, 2205-17 (2004).

48. Coste, B. *et al.* Gain-of-function mutations in the mechanically activated ion channel PIEZO2 cause a subtype of Distal Arthrogryposis. *Proc Natl Acad Sci U S A* **110**, 4667-72 (2013).

49. Alper, S.L. Genetic Diseases of PIEZO1 and PIEZO2 Dysfunction. *Curr Top Membr* **79**, 97-134 (2017).

50. McMillin, M.J. *et al.* Mutations in PIEZO2 cause Gordon syndrome, Marden-Walker syndrome, and distal arthrogryposis type 5. *Am J Hum Genet* **94**, 734-44 (2014).

51. Schrauwen, I. *et al.* Variants affecting diverse domains of MEPE are associated with two distinct bone disorders, a craniofacial bone defect and otosclerosis. *Genet Med* **21**, 1199-1208 (2019).

52. Tomas-Roca, L. *et al.* De novo mutations in PLXND1 and REV3L cause Mobius syndrome. *Nat Commun* **6**, 7199 (2015).

53. Glass, G.E. *et al.* Poland-Mobius syndrome: a case report implicating a novel mutation of the PLXND1 gene and literature review. *BMC Pediatr* **22**, 745 (2022).
