# Supplementary material for: Systematic phenotype and genotype characterization of Moebius syndrome
Source: Genet Med Open. 2025 May 19;3:103437. doi: 10.1016/j.gimo.2025.103437 (PMC12256340; doi:10.1016/j.gimo.2025.103437)
Supplement: Supplementary Figures [file mmc3.pdf]

## Supplementary Figure 1.

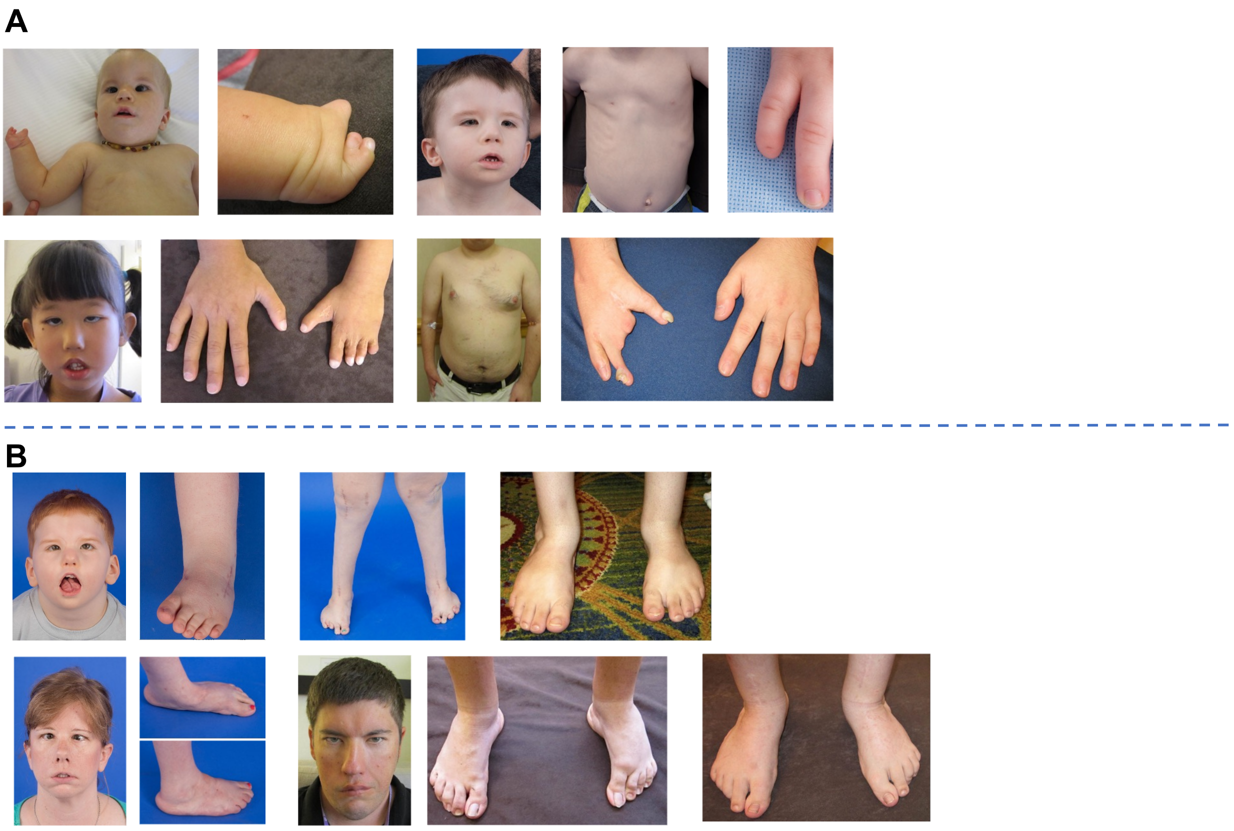


**Supplementary Fig. 1: MBS associated features.** (**A)** Individuals with Moebius syndrome with pectoralis muscle hypoplasia, with hypoplastic or displaced nipple. This may or may not present with variable degree of ipsilateral hand and digit anomalies, including brachymelia, absent/small hand and/or oligodactyly, brachy and/or symbrachydactyly, thumb hypoplasia, camptodactyly (MBS-Poland). (**B**) Representative cases of individuals with Moebius syndrome and unilateral or bilateral talipes equinovarus (clubfoot).

**Supplementary Figure 2.**

A

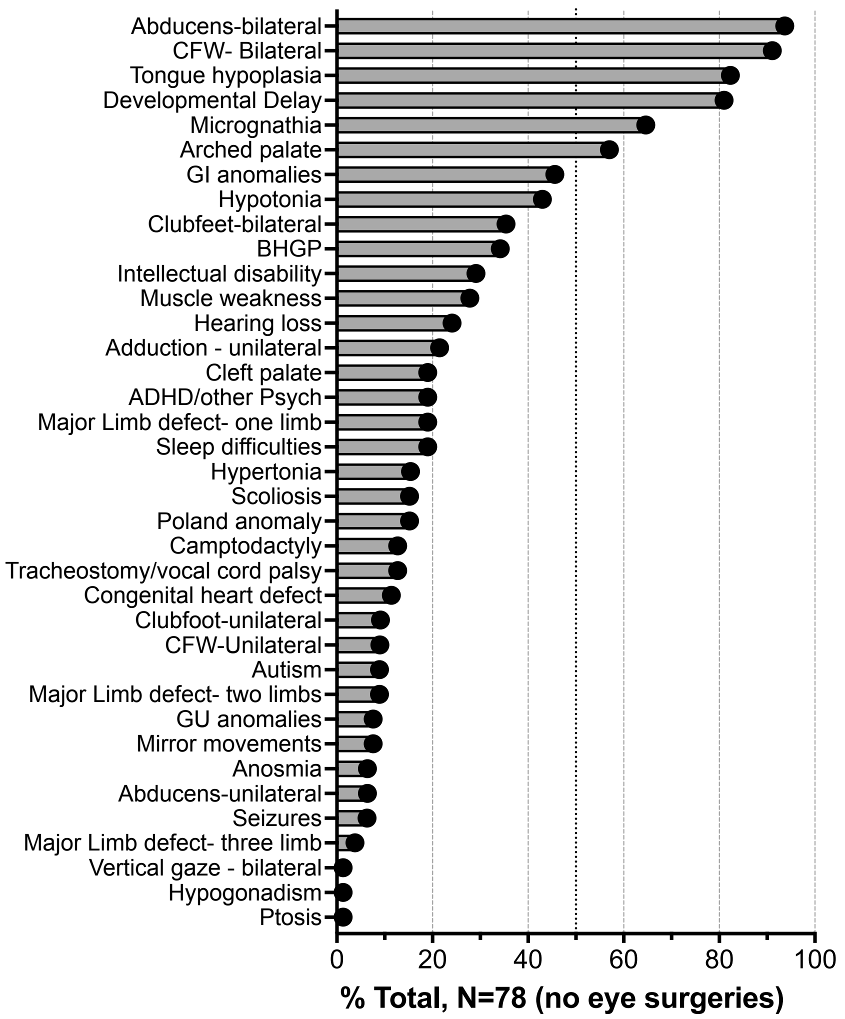


B

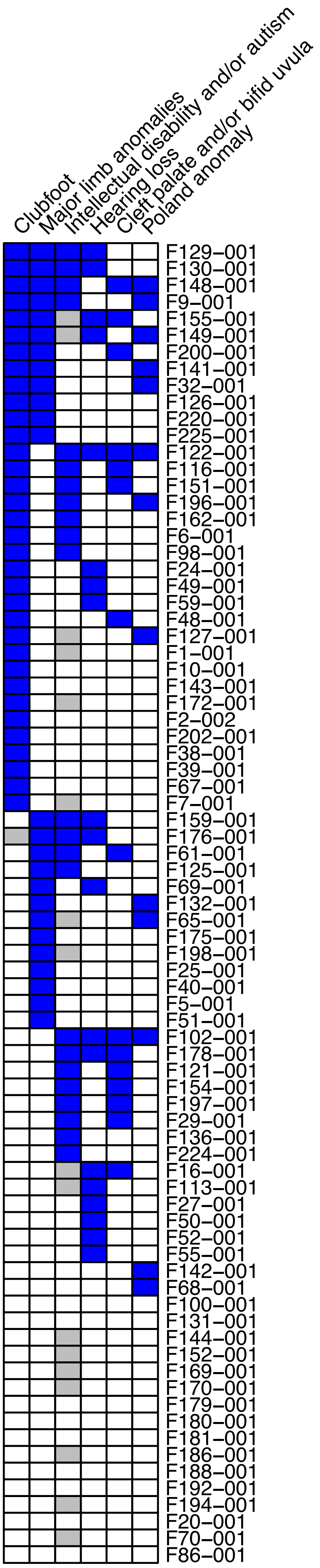


**Supplementary Fig. 2: Phenotypic spectrum of MBS in probands without prior eye surgery. (A)** Incidence of key phenotypic features in the cohort (n=78) sorted by frequency. **(B)** Unsupervised clustering of participants given presence of six features (clubfoot, major limb anomalies, intellectual disability and/or autism, hearing loss, cleft palate or bifid uvula, and Poland anomaly/pectoralis hypoplasia, with features listed in highest to lowest frequency for the affected patients who had not had prior eye surgeries (N=79). Phenotype data is visualized using the pheatmap R package.

ADHD-attention deficit hyperactivity disorder; BHGP-bilateral horizontal gaze paresis; CFW-congenital facial weakness; GI-gastrointestinal; GU-genitourinary; trach- tracheostomy.

**Supplementary Figure 3.**

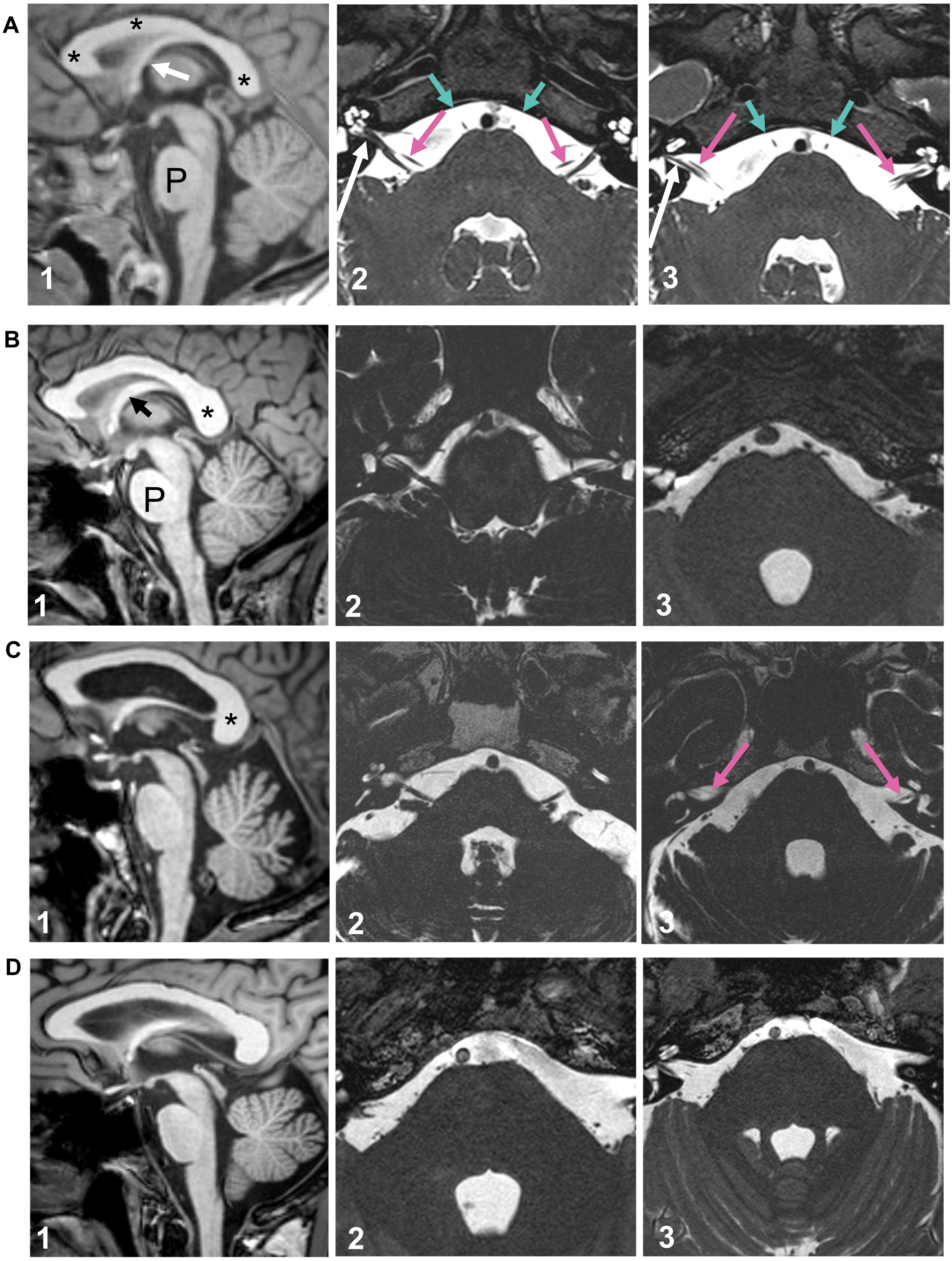


**Supplementary Fig. 3**: **Brain magnetic resonance imaging findings in MBS** (**A**) Control images provided here for comparison to B-D. (**B**) MR images from F2-001, a 38-year-old woman with MBS and bilateral talipes equinovarus. **1**. Sagittal MPRAGE MR image showing variant anatomy of the corpus callosum with a thickened splenium (black asterisk) and fornices (short black arrow). The pons appears minimally shortened in height and slightly more rounded superiorly than normal. **2**. Axial 3D T2 weighted MR image shows absence of CN VI bilaterally. **3.** Axial 3D T2 volume isotropic turbo spin-echo acquisition (VISTA) MR image shows absence of CN VII bilaterally. The threadlike, curving, tubular structures on either side of the basilar artery (white asterisks) in 2 and 3 are vessels. (**C**) MR images from F104-001, a 47-year-old man with MBS and bilateral brachysyndactyly. **1**.Sagittal MPRAGE MR image shows variant anatomy of the corpus callosum with a slightly prominent splenium (asterisk). The pons appears normal. **2**. Axial 3D T2 VISTA MR image with non-visualization of the cisternal segments of CN VI and VII bilaterally. **3**. Axial 3D T2 VISTA MR image with meatal segments of CN VII faintly visualized more laterally within each IAC (magenta arrows). (**D**) MR images from F117-001, a 54-year-old man with MBS and mirror movements. **1.** Sagittal MPRAGE MR image shows normal anatomy of the corpus callosum and pons. **2**. Axial 3D T2 weighted MR image shows a small structure in the expected location of CN VI – either a hypoplastic nerve or small vessel. Left CN VI is not seen. **2 and 3**. Axial 3D T2 weighted MR image shows that the meatal segments of CN VII are absent bilaterally. Curvilinear hypointense structures are loops of the anterior inferior cerebellar arteries.

**Supplementary Figure 4.**

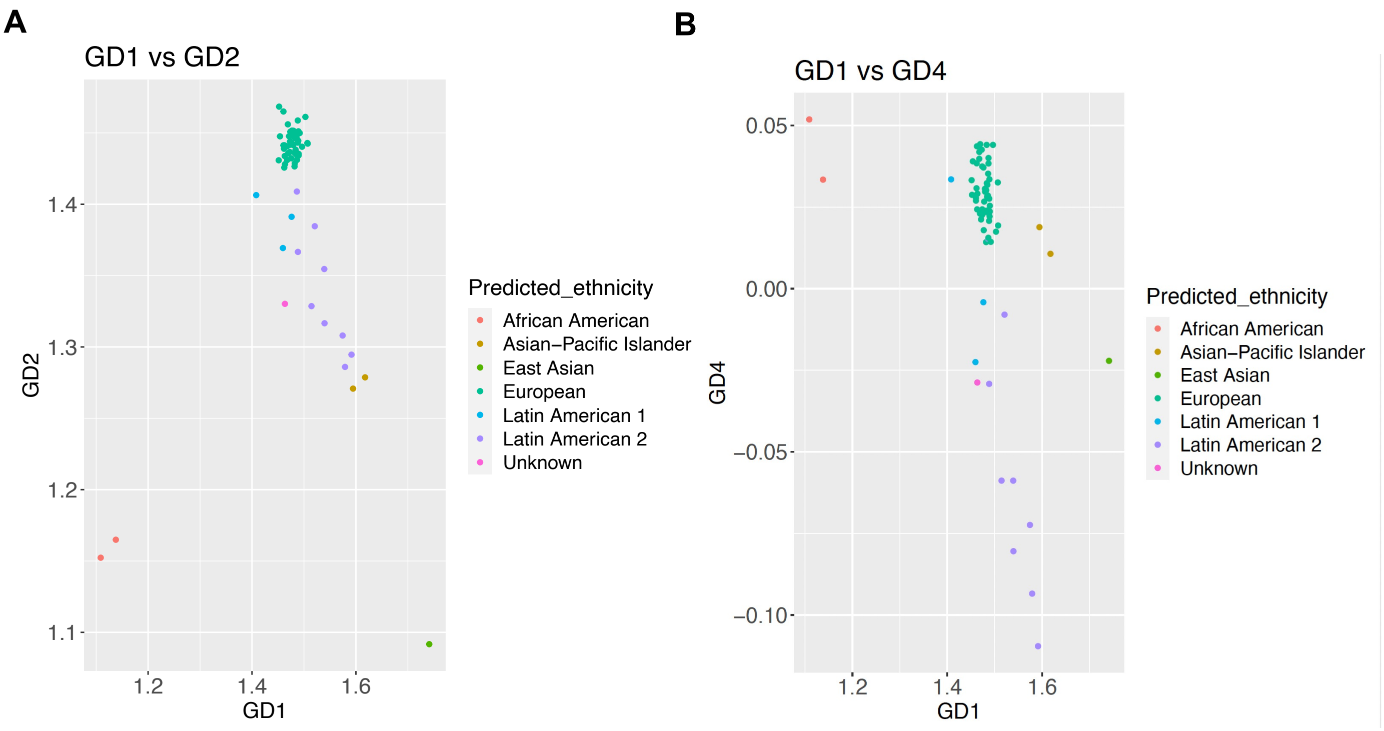


**Supplementary Fig. 4: Ancestry prediction of sequenced probands.** Genetic distances were calculated using the GRAF-pop method across the 67 probands with exome or genome sequencing data. Ancestries were assigned using default options of the software. **(A)** GD1 vs GD2; **(B)** GD1 vs GD4. GRAF-pop calculates multiple genetic distances of each subject from three reference populations (European, East Asian, and African) using fingerprint SNPs. Ancestry is inferred from the genetic distance scores from each reference population. GD1=Genetic distance 1; GD2=Genetic distance 2; GD3: Genetic distance 3; GD4: Genetic distance4; Predicted ethnicity=estimated ethnicity based on the SNP genotypes using GRAF-pop.

**Supplementary Figure 5.**

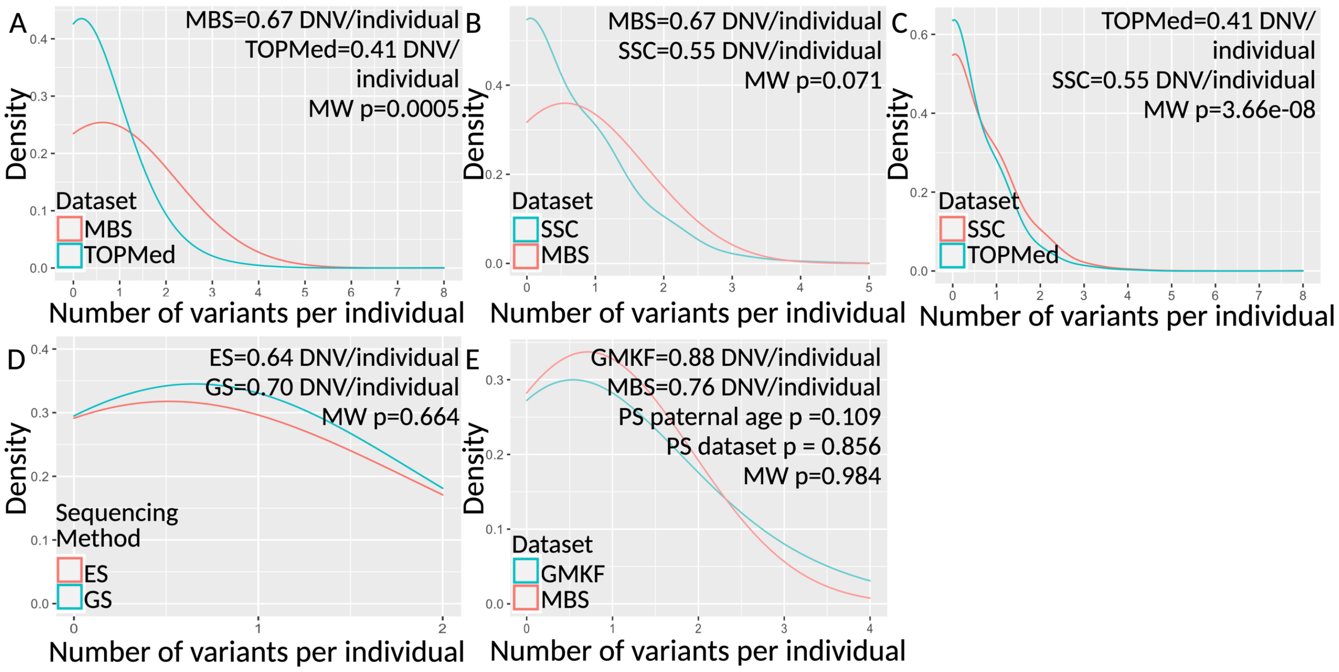


**Supplementary Fig. 5: De novo variant analysis.** The relative rates of rare (MAF<0.00001) coding or splice site *de novo* SNVs per individual were compared and schematized as density plots denoting proportions of each group that have various numbers de novo variants, using the following comparison groups: **(A)** 58 MBS trios (29 with WES, 29 with WGS) versus WGS of 1,449 TOPMed trios; **(B)** 58 MBS trios (29 with WES, 29 with WGS) versus WES of 1,789 SSC control trios; **(C)** WGS of 1,449 TOPMed trios versus WES of 1,789 SSC control trios; **(D)** MBS trios sequenced by WES (n=29) versus WGS (n=29); **(E)** WGS of 31 MBS trios versus 40 non-MBS GMKF control trios sequenced and processed in parallel. Mean numbers of rare (MAF<0.00001) coding or splice site *de novo* SNVs per individual are denoted as "DNV/individual." De novo variant rates were compared between datasets by the Mann-Whitney two-sided U test (A-E) and by Poisson regression using the dataset (MBS or non-MBS GMKF) and paternal age at conception as covariates (E). WES=whole exome sequencing, WGS=whole genome sequencing, MBS=Moebius syndrome trios, TOPMed=TOPMed cohort trios, SSC=Simons Simplex Consortium control trios, GMKF=Gabriella Miller Kids First control trios, MW=Mann-Whitney 2-sided U test, PS=Poisson regression. Figure schematic assembled with Biorender.com; data analysis performed in RStudio.
